# Agdc1p – a Gallic Acid Decarboxylase Involved in the Degradation of Tannic Acid in the Yeast *Blastobotrys (Arxula) adeninivorans*

**DOI:** 10.3389/fmicb.2017.01777

**Published:** 2017-09-15

**Authors:** Anna K. Meier, Sebastian Worch, Erik Böer, Anja Hartmann, Martin Mascher, Marek Marzec, Uwe Scholz, Jan Riechen, Kim Baronian, Frieder Schauer, Rüdiger Bode, Gotthard Kunze

**Affiliations:** ^1^Leibniz Institute of Plant Genetics and Crop Plant Research Gatersleben, Germany; ^2^Department of Genetics, Faculty of Biology and Environmental Protection, University of Silesia Katowice, Poland; ^3^Department of Microbiology, School of Biological Sciences, University of Canterbury Christchurch, New Zealand; ^4^Institute of Microbiology, University of Greifswald Greifswald, Germany

**Keywords:** biodegradation, aromatic acids, gallic acid, antimicrobial plant substances, fungi

## Abstract

Tannins and hydroxylated aromatic acids, such as gallic acid (3,4,5-trihydroxybenzoic acid), are plant secondary metabolites which protect plants against herbivores and plant-associated microorganisms. Some microbes, such as the yeast *Arxula adeninivorans* are resistant to these antimicrobial substances and are able to use tannins and gallic acid as carbon sources. In this study, the *Arxula* gallic acid decarboxylase (Agdc1p) which degrades gallic acid to pyrogallol was characterized and its function in tannin catabolism analyzed. The enzyme has a higher affinity for gallic acid (K_m_ −0.7 ± 0.2 mM, k_cat_ −42.0 ± 8.2 s^−1^) than to protocatechuic acid (3,4-dihydroxybenzoic acid) (K_m_ −3.2 ± 0.2 mM, k_cat_ −44.0 ± 3.2 s^−1^). Other hydroxylated aromatic acids, such as 3-hydroxybenzoic acid, 4-hydroxybenzoic acid, 2,3-dihydroxybenzoic acid, 2,4-dihydroxybenzoic acid and 2,5-dihydroxybenzoic acid are not gallic acid decarboxylase substrates. *A. adeninivorans* G1212/YRC102-AYNI1-AGDC1, which expresses the *AGDC1* gene under the control of the strong nitrate inducible *AYNI1* promoter achieved a maximum gallic acid decarboxylase activity of 1064.4 U/l and 97.5 U/g of dry cell weight in yeast grown in minimal medium with nitrate as nitrogen source and glucose as carbon source. In the same medium, gallic acid decarboxylase activity was not detected for the control strain G1212/YRC102 with *AGDC1* expression under the control of the endogenous promoter. Gene expression analysis showed that *AGDC1* is induced by gallic acid and protocatechuic acid. In contrast to G1212/YRC102-AYNI1-AGDC1 and G1212/YRC102, *A. adeninivorans* G1234 [Δ*agdc1*] is not able to grow on medium with gallic acid as carbon source but can grow in presence of protocatechuic acid. This confirms that Agdc1p plays an essential role in the tannic acid catabolism and could be useful in the production of catechol and *cis,cis*-muconic acid. However, the protocatechuic acid catabolism via Agdc1p to catechol seems to be not the only degradation pathway.

## Introduction

The non-conventional, non-pathogenic, imperfect, dimorphic yeast *Arxula adeninivorans* (syn. *Blastobotrys adeninivorans*) is a fungal organism with great biotechnological potential due to its wide substrate spectrum and robustness against osmotic, salt and temperature stresses (Middelhoven et al., 1984; Wartmann et al., 1995; Malak et al., 2016). It grows in harsh environments, tolerates up to 20% NaCl in the medium, endures temperatures of ≤48°C. It is also able to use a variety of tannins including tannic acid and hydroxylated aromatic acids as carbon sources (Wartmann et al., 1995; Tag et al., 1998; Yang et al., 2000; Böer et al., 2009a). To date, this research has focused on the wildtype strain, *A. adeninivorans* LS3 that was isolated from wood hydrolysate in Siberia (Gienow et al., 1990). In 2014, the complete genome of *A. adeninivorans* LS3 was sequenced and genome analysis revealed the presence of two potential tannin acyl hydrolases (Atan1p, Atan2p) as well as gallic acid decarboxylase (Agdc1p) (Kunze et al., 2014).

Tannins are part of almost all types of plant tissues and their hydrolysis releases glucose and gallic acid. Gallic acid belongs to the hydroxylated aromatic acid compounds, which share primarily antifungal and bacteriostatic properties and are toxic for most microbes at very low concentrations (Sikkema et al., 1995). Gallic acid recently became a useful compound in pharmaceutical and chemical industries because of its antifungal, bacteriostatic, anticancer, antimelanogenic, and antioxidant properties (Badhani et al., 2015).

A large amount of gallic acid is produced in the food and agriculture industries, which has contributed to a remarkable increase of phenolic compounds, mostly toxic agents, in the environment. Unfortunately, di- and trihydroxylated aromatic acids such as gallic acid and protocatechuic acid are difficult to degrade and often accumulate in water and soil, which is resulting in additional environmental pollution (Zhang et al., 2014). Before there can be an attempt to increase the efficiency of bioremediation processes, the microorganisms and the enzymes that degrade these pollutants need to be investigated.

Many bacteria and filamentous fungi have been characterized as organisms able to degrade hydroxylated aromatic acids by enzymatic degradation. These microorganisms developed several different ways to degrade these aromatic compounds. One option is the decarboxylation of a carboxyl group attached to the aromatic ring, which is catalyzed by enzymes from the decarboxylase family (Grant and Patel, 1969; Yoshida et al., 1982; Wright, 1993; O'Donovan and Brooker, 2001; Hashidoko et al., 2002; Mukherjee and Banerjee, 2004). In most cases, these decarboxylases are encoded by inducible genes, which ensure that the synthesis of the enzymes only occurs in the presence of their substrates. However, only a few of these enzymes have been characterized to date because they exhibit high oxygen sensitivities and are relatively unstable (Yoshida et al., 1982; Nakajima et al., 1992; Zeida et al., 1998; Jiménez et al., 2013).

The general strategy of aerobic degradation of aromatic compounds is the cleavage of the aromatic ring with the degradation products being channeled into the central metabolism. Usually the compounds go through *ortho* or *meta* pathways and are completely utilized (Williams and Sayers, 1994). However, there are microorganisms which can non-oxidatively decarboxylate gallic acid but the pathway results in death of the cell because of a lack in further degradative enzymes (Nakajima et al., 1992; Zeida et al., 1998; Rodríguez et al., 2008).

The decarboxylation of gallic acid results in the production of pyrogallol. This phenol derivate is very sensitive to oxygen and is used in many industrial applications, for example as a developing agent in photography and in cosmetic products, such as in hair dying agents.

The tannin acyl hydrolase 1 (Atan1p) has been already characterized and described as the enzyme which catalyzes the first reaction step in tannin degradation by *A. adeninivorans* LS3 (Böer et al., 2009a). It was described as extracellular enzyme, induced by tannins or gallic acid. The transformation of gallic acid and protocatechuic acid by *A. adeninivorans* LS3 wildtype strain have also been investigated (Sietmann et al., 2010). However, it is still not known if the degradation pathway is common for all hydroxylated aromatic acids and the role of gallic acid decarboxylase is currently unclear.

In this study, the *Arxula* gallic acid decarboxylase gene, *AGDC1*, was identified, isolated and expressed using a strong promoter in *A. adeninivorans*. The recombinant enzyme was purified, characterized and in addition, its function in the yeast cell determined. Growth of the *AGDC1* expression yeast strain and a Δ*agdc1* gene disruption mutant on different aromatic acids was studied to elucidate the role of the gene product in *A. adeninivorans*.

## Materials and methods

### Strains and culture conditions

*Escherichia coli* strains XL1 blue [r*ecA1, endA1, gyrA96, thi-1, hsdR17, supE44, relA1, lac* [F'proABlacl q Z DM15 Tn10 (Tetr)], from Invitrogen (Grand Island, NY, USA) or DH5α [F– Φ80d*lac*ZΔM15 Δ(*lac*ZYA-*arg*F) U169, deoR, *rec*A1, *end*A1, *hsd*R17 (r_K_−, m_K_+) *pho*A *sup*E44, λ^−^, *thi*-1, *gyr*A96 *rel*A1] were used as host strains for bacterial transformation and plasmid isolation. Both strains were cultured in Luria Bertani medium (LB - Sigma, USA) supplemented with 50 mg/l ampicillin (AppliChem, Germany) or 50 mg/l kanamycin (Roth, Germany).

*Arxula adeninivorans* G1212 (*aleu2 ALEU2::atrp1*) (Steinborn et al., 2007) and the wildtype strain *A. adeninivorans* LS3 (Kunze and Kunze, 1994), originally isolated from wood hydrolysate in Siberia (Russia), were used as experimental strains. Both strains are deposited in the yeast collection of the Department of Biology of the University of Greifswald (SBUG, Germany). They were cultivated in shaking flasks at 30°C, 180 rpm either under non-selective conditions in complex medium (YEPD-yeast extracts-peptone-dextrose) or in yeast minimal medium (YMM) supplemented with 43.5 mM NaNO_3_ as nitrogen source and 20 g/l glucose as carbon source (YMM-glucose-NaNO_3_) (Tanaka et al., 1967; Rose et al., 1990).

### Transformation procedures and isolation of nucleic acids

Transformations of *E. coli* and *A. adeninivorans* were performed according to Böer et al. (2009a). Plasmid DNA isolation and DNA restriction were carried out as described by Wartmann et al. (2002).

### Construction of *AGDC1* expression plasmids and generation of transgenic *A. adeninivorans* strains

For *AGDC1* overexpression, the *AGDC1* ORF (PRJEB19393) with a HisTag encoding region at the 3′-end (*AGDC1-6H*) was amplified from genomic DNA of *A. adeninivorans* LS3 in a PCR reaction using primers that incorporated flanking *Eco*RI and *Not*I cleavage sites: primer AGDC1-1-6H-5′-**GAA TTC** ATG ACT ACT TCC TAC GAG CCC TGG-3′ (primer nucleotide positions 1-20, *Eco*RI restriction site is in bold type and underlined); primer AGDC1-2-6H 5′-**GCG GCC GC**T TAG TGG TGG TGA TGA TGG TGC CAG TGG AGG TCA ATC TCC T-3′ (primer nucleotide positions 674–693, HisTag encoding region underlined, *Not*I restriction site is in bold type and underlined). The resulting *Eco*RI-*Not*I flanked AGDC1-6H ORF was inserted into the plasmid pBS-AYNI1-PHO5-SS between the *A. adeninivorans* derived *AYNI1* promoter and the *S. cerevisiae PHO5* terminator (Böer et al., 2009c). The *AYNI1* promoter *- AGDC1-6H* gene *- PHO5* terminator flanked by *Spe*I–*Sac*II restriction sites expression modules were inserted into the plasmid Xplor2.2 to generate Xplor2.2-AYNI1-AGDC1-6H-PHO5 (Böer et al., 2009b). The resulting plasmids contained fragments of 25S rDNA, which are interrupted by the selection marker module (*ALEU2* promoter-*ATRP1m* gene-*ATRP1* terminator), the AGCD1 expression module and an *E. coli* resistance marker and replicator. To prepare the cassettes for yeast transformation, Xplor2.2-AYNI1-AGDC1-6H-PHO5 and the control plasmid Xplor2.2 lacking AGDC1 expression module were digested with *Asc*I (YRC) or *Sbf* I (YIC) to remove the *E. coli* sequences including the resistance marker. The resulting restriction products YRC102-AYNI1-AGDC1-6H, YIC102-AYNI1-AGDC1-6H and YRC102 (control) were used to transform *A. adeninivorans* G1212.

Yeast transformants were selected by tryptophan auxotrophy in YMM-glucose-NaNO_3_. The cells were stabilized by passaging on selective (YMM-glucose-NaNO_3_) and non-selective (YEPD) agar medium, to attain a high level of protein production (Klabunde et al., 2003).

### Construction of Δ*agdc1* gene disruption mutant

To create disruption mutants, the gene disruption cassette contained 1,019 bp up and 1,032 bp down the ORF of *AGDC1* as well as the ATRP1m selection marker module was constructed. All fragments were amplified by PCR (up and down ORF overlapped fragments using chromosomal DNA of *A. adeninivorans* LS3 as the template and ATRP1m selection marker module using plasmid pBS-ALEU2-TRP1m as the template-Steinborn et al., 2007). Primers used for fragment amplification were created with additional 15 bp overlapping sides (Table 1A). This strategy allowed ligation of fragments in one step by using an In-Fusion Cloning Kit (TaKaRa Clontech, USA Inc.). The resulting construct was amplified in *E. coli* DH5α. Finally, the complete construct covering 1019 bp in front of the *AGDC1* gene, the ATRP1m selection marker module and 1032 bp behind the *AGDC1* was amplified using the primers 5′-AAATTCTTCTACAGGAAGTCAGG-3′ and 5′-ATTGTTATGCACTATTCGTTAACGG-3′. The resulting 3,548 bp PCR-product was used to transform *A. adeninivorans* G1212 (Böer et al., 2009a).

**Table 1:** **(A)** Primers used for construction of deletion mutant strain G1234 [Δ*agdc1*]. **(B)** Primers used for analysis of *AGDC1* expression levels.

**(A) Primers for construction of *A. adeninivorans* G1234 [Δ*agdc1*]**		
**Primer**		**Sequence (5′ → 3′)**
01.1000bp ΔAGDC left		GTTTTAATTACAAAAAGCTAAATTCTTCTACAGGAAGTCAGG
02. Primer right 1 ΔAGDC		TGTACAATGTTTCTTTCTTGTCTG
03. Marker left ΔAGDC		AAGAAACATTGTACATTTCAATCGACGATTGCAATTGAC
04. Marker right 1 ΔAGDC		GAGTCTGTATTGAAGCTTAGCTTG
05. Primer left 1 ΔAGDC		CTTCAATACAGACTCACAGAACTAGAATATATAACAATGTTATTAAA
06.1000bp ΔAGDC right		TTAGTTAAAAGCACTCCATTGTTATGCACTATTCGTTAACGG
**(B) Primers for nested quantitative RT PCR**		
**Reaction step**	**Primer**	**Sequence (5′ → 3′)**
Step1 cDNA synthesis	(dT) 15V-RTA	TGA CAG GAT ACC ATA CAG ACA CTA TTT TTT TTT TTT TTT V
Step 2 Second PCR synthesis	RTA-1 rv	TGA CAG GAT ACC ATA CAG ACA C
	AGDC1-V fw	TGC TGT GGC TCA GGC TAA TC
	TFC1-3 fw	TGA AGA AGA GCA CCA AGC A
	ALG9-7	TGGTATCGGTCGCATTCT
Step 3 Second PCR synthesis	RTA rv	TGA CAG GAT ACC ATA CAG ACA CTA
	AGDC1-III fw	ATG TAC GAC GCC ATG AAG GA
	TFC1-1 fw	ACA ACA AGA TGA AAA CGC
	ALG9-8	TCAATTGCAGTGGACTGACTA

### Microscopic analysis

The phenotype of *A. adeninivorans* G1212/YRC102, G1212/YRC102-AYNI1-AGDC1-6H and G1234 [Δ*agdc1*] was investigated. For this purpose, strains were cultivated on agar plates containing YMM-glucose-NaNO_3_ at 30°C for 48 h. Colony morphology was investigated using a VHX-5000 Digital Microscope (KEYENCE Deutschland GmbH, Neu-Isenburg, Germany). For detailed analysis, a FESEM S 4100 device (Hitachi High-Technologies Europe GmbH, Krefeld, Germany) was used. Fragments of agar plates containing colonies were dried in 30°C, attached to carbon-coated aluminum sample blocks and gold-coated in an Edwards S150B sputter coater (Edwards High Vacuum Inc., Clevedon, United Kingdom).

### Assay for determination of gallic acid decarboxylase activity

Gallic acid decarboxylase activity was assayed by following gallic acid or protocatechuic acid biotransformation during the enzymatic reaction in 50 mM potassium phosphate buffer pH 6.2. The reactions were carried out in 96-well UV-transparent microtiter plates (Greiner Bio-One GmbH) in triplicates, in a total volume of 100 μl. Reaction was started by adding 0.5 mM substrate solution to the enzyme and monitored 15 min at 40°C, 259 nm (ε = 7100 [1/M/cm]) and 288 nm (ε = 1,570 [1/M/cm]) for gallic acic and protocatechuic acid respectively (Krumholz et al., 1987). Blank values were established by assaying using water instead of the enzyme. One Unit (1 U) of enzyme activity was defined as the amount of enzyme required to decarboxylate 1 μmol gallic acid to pyrogallol or 1 μmol protocatechuic acid to catechol per min at 40°C, pH 6.2.

Gallic acid decarboxylase activity with 3-hydroxybenzoic acid, 4-hydroxybenzoic acid, 2,3-dihydroxybenzoic acid, 2,4-dihydroxybenzoic acid, 2,5-dihydroxybenzoic acid as substrates were measured by GC-MS. 470 μl 1 mg/ml substrate in 50 mM potassium phosphate buffer pH 6.2 and 30 μl purified Agdc1p were incubated at 40°C for 0, 5, 15, 25, and 35 min. The blank for the reaction was assayed using boiled enzyme (15 min, 95°C). The reactions were stopped by increasing the temperature for 95°C for 15 min. The pH of the samples was adjusted to 2 with HCl and the compounds extracted using organic solvent MTBS (Sigma-Aldrich, USA). Extracts were lyophilized and resuspended in 400 μl pyridine and 100 μl BSTFA (Sigma-Aldrich, USA) and incubated overnight at 60°C. GC/MS measurements were done according to method described in “Analysis of supernatant from strains growing on YMM-NaNO_3_ supplemented with different carbon sources.”

### Agdc1-6hp analysis

Agdc1-6hp was purified by column chromatography on HisTrap FF (1 ml) Novagen, USA) in 20 mM Tris, 0.5 mM NaCl pH 7.9, and 5 mM imidazole as binding buffer and 20 mM Tris, 0.5 mM NaCl pH 7.9, and 1 M imidazole as elution buffer. Finally, the purified protein was desalted with PD10 columns (GE Health Care Europe GmbH, Germany).

The indicative molecular mass determination of native Agdc1-6hp was done by gel filtration using Superdex™ 200 (Amersham Biosciences, UK). The flow rate was 1 ml/min and fractions of about 1 ml were collected for 182 min (buffer: 50 mM Tris pH 8 + 0.15 M NaCl). A calibration curve was constructed using blue dextran, ferritin, catalase, bovine albumin, RNAse A and vitamin B12 as standards.

The K_m_ value for gallic acid and protocatechuic acid were determined as described in “Assay for determination of gallic acid activity.” All measurements were done in triplicate and Michaelis-Menten and Hanes plots were constructed.

The Agdc1p concentration was determined using a Coomassie stained SDS-PAGE for the calculation of k_cat_ (Kunze et al., 1998).

### Protein analysis

SDS-PAGE and Western blot analysis were performed as described by Kunze et al. (1998). The antibodies used for Western blot analysis were anti-His-tag specific primary antibody produced in rabbit (Sigma-Aldrich, USA) and secondary antibody anti-mouse IgG alkaline phosphatase conjugate produced in goat (Sigma-Aldrich, USA). The staining procedure was done by membrane incubation with NBT/BCIP substrate (Roche Diagnostics, Switzerland).

The dye-binding method of Bradford was used for protein quantification (BIO-RAD, USA), using bovine serum albumin as the standard (Bradford, 1976).

### Quantitative reverse transcriptase PCR analysis (qRT PCR)

*Arxula adeninivorans* G1212/YRC102 cells were grown in YMM-glucose-NaNO_3_ for 24 h at 30°C and 180 rpm. Afterwards cells were harvested (3,220 × g at 4°C), washed with YMM-NaNO_3_ and resuspended in YMM-NaNO_3_ containing 0.2% glucose or hydroxylated aromatic acids. 2 ml of culture samples were collected after 0, 4, 8, 24, and 48 h. Cells were pelleted (2,300 × g, 4°C), and disrupted mechanically using silica beads and a Mixer Mill MM400 (RETSCH, Germany) operating for 3 min, vibrational frequency of 30/s. Total RNA was isolated using RNeasy Mini Kit (Qiagen, Germany) as described by the manufacturer. RNA concentration was analyzed using NanoDrop2000c spectrophotometer (Thermo Fisher, Germany). cDNA was synthesized using RevertAid H Minus First Strand cDNA Synthesis Kit (Fermentas, Germany) with an Oligo(dT)15V-RTA primer. For the analysis of temporal gene expression patterns a nested qRT-PCR assay was applied (Worch and Lemke, 2017). The first PCR synthesis was made with cDNA-template (10 cycles) using gene-specific primers and RTA1 primer (Table 1B). The PCR product was diluted 1:500 and amplified in the presence of SYBR Green fluorescent dye (Power SYBR® Green PCR Master Mix, Applied Biosystems, Foster City, CA, USA) using ABI 7900HT Fast Sequence Detection System (Applied Biosystems) with gene-specific primers 2 or 4 and RTA primer (Table 1B). *TFCI* and *ALG9* were used as reference (housekeeping) genes (Rösel and Kunze, 1995; Teste et al., 2009). The calculations were done using ΔΔc_t_-method (Livak and Schmittgen, 2001).

### Microarray design and hybridization for gene expression analysis

The gene expression profiling was performed using a microarray produced by Agilent Technologies in 8 × 60 k format. The microarray was based on 6,025 annotated chromosomal sequences and 36 putative mitochondrial gene oligos and was designed using Agilent Technologies eArray software (https://earray.chem.agilent.com; design number 035454). Depending on the sequence length of the genes, up to 10 60-mers per gene were created, resulting in a total of 56,312 *A. adeninivorans* specific oligos.

*A. adeninivorans* LS3 was cultivated overnight in YMM-glucose-NaNO_3_. Cells were then pelleted (3,220 × g at 20°C), and shifted to YMM-NaNO_3_ containing a mixture of 0.5% gallic acid plus 1% glucose or 1% glucose only. After 15 min, 30 min, 2 and 5 h of cultivation at 30°C and 180 rpm, cells were harvested and total RNA was isolated using RNeasy Mini Kit (Qiagen, Germany). The samples were labeled and the microarray hybridized according to the Agilent Technologies “One-Color Microarray-Based Gene Expression Analysis (v6.5)” instructions. The R package limma was used for microarray data analysis (Smyth, 2005). The background of raw expression data was corrected by “normexp” and normalized between arrays using “quantile.” Differentially expressed genes were detected by fitting a linear model to log_2_-transformed data by an empirical Bayes method (Smyth, 2004). The Bonferroni method was used to correct for multiple testing.

### Analysis of supernatant from strains growing on YMM-NaNO_3_ supplemented with different carbon sources

Quantities of glucose, hydroxylated aromatic acids, as well as various phenol derivatives were measured by GC-MS (Clarus SQ 8 GC Mass Spectrometers). Samples were previously extracted and derivatized. For this purpose, 2 ml cultures were centrifuged for 10 min, 16,000 × g, 4°C, 500 μl supernatant was adjusted to pH 2 with HCl and compounds were extracted with 1 ml MTBS (Sigma-Aldrich, USA). Extracts were lyophilized in a Freeze Dryer, Alpha 1-4 LSC plus (Christ), resuspended in 400 μl pyridine, 100 μl BSTFA (Sigma-Aldrich, USA) was added and samples were incubated overnight at 60°C. 1 μl sample was injected into an Elite-5MS column, Length 30 m, I.D 0.25 mm, 25 μm (Elite, USA). The injection was performed with a split speed 10 ml/min. The temperature was held at 60°C for 1 min and was then ramped up to 230°C at 15°C/min and held at this temperature for 11.33 min. The peak areas (triplicates) were calculated by TurboMass 6.1 using data from external quantification standards assayed four times.

## Results

### Identification of the *A. adeninivorans AGDC1* gene encoding gallic acid decarboxylase (Agdc1p)

A putative *gallic acid decarboxylase* gene (ARAD1C45804g) was annotated in the genome of *A. adeninivorans* LS3. The gene, *Arxula gallic acid decarboxylase 1* (*AGDC1*), is localized on chromosome *Arad1C*. The open reading frame is a 696 bp encoding a protein with 232 amino acids. The predicted subunit molecular mass of Agdc1p is 27.3 kDa. Protein analysis according to the SignalP (version 4.1) program (http://www.cbs.dtu.dk/services/SignalP/) predicted the absence of a secretion signal sequence, which was corroborated by cytoplasmic localization in cell fractionation experiments (data not shown).

**Figure 1:**
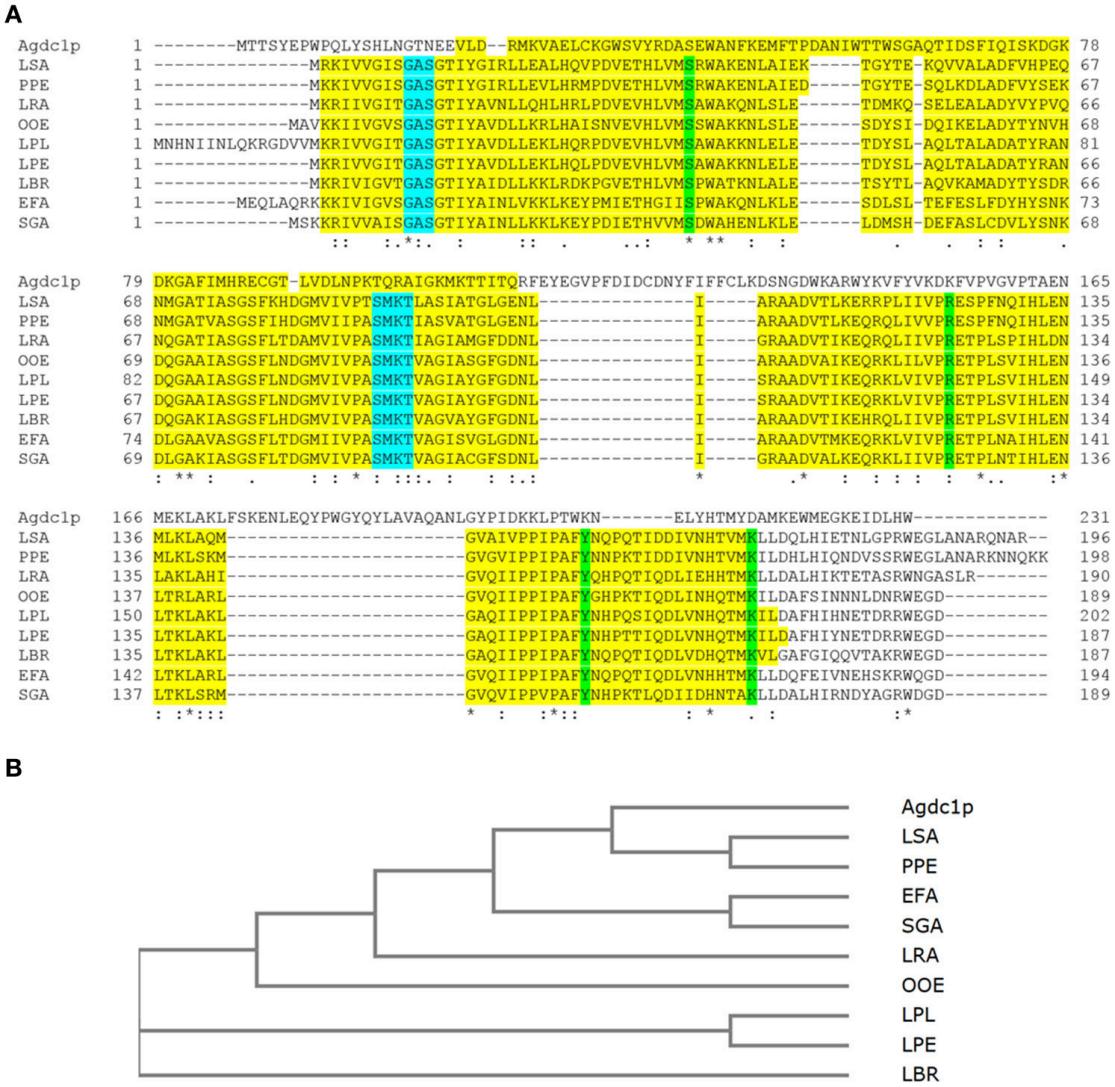
**(A)** Multiple alignment of Agdc1p sequence to subunit B from *Lactobacillus plantarum* ATCC 14917^T^ (LPL) (D7V849) and other putative gallic acid decarboxylases from lactic acid bacteria *Lactobacillus pentosus* KCA1 (LPE) (I9L531), *Lactobacillus brevis* ATCC 367 (LBR) (Q03P27), *Lactobacillus rhamnosus* HN001 (LRA) (B5QPH7), *Lactobacillus sakei* 23K (LSA) (Q38Y44), *Enterococcus faecium* DO (EFA) (Q3Y2U4), *Oenococcus oeni* PSU-1 (OOE) (A0NKP0), *Pediococcus pentosaceus* ATCC 25745 (PPE) (Q03HH4), and *Streptococcus gallolyticus* UCN34 (SGA) (D3HEZ0). Alignment was done using ClustalOmega. The domains are highlighted in yellow; binding sites in green and nucleotide binding sites in blue. **(B)** The phylogenetic tree was constructed by the neighbor joining method of Agdc1p to subunit B of selected lactic bacteria without distance corrections. An asterisk (^*^) indicates positions which have a single, fully conserved residue, a colon (:) indicates conservation between groups of strongly similar properties, and a period (·) indicates conservation between groups of weakly similar properties.

In *Lactobacillus plantarum* WCFS1 the enzyme comprises three subunits. There are several conserved domains that are fundamental for enzyme catalytic activity and structure were found in the Agdc1p sequence such as subunit B which is homologous with *Lactobacillus plantarum* WCFS1 gallic acid decarboxylase and putative gallic acid decarboxylase protein sequences from other lactic bacteria described by Jiménez et al. (2013) (Figure 1A).

A phylogenetic tree between Agdc1p and gallic acid decarboxylase from *L. plantarum* as well as the putative gallic acid decarboxylase protein sequences from lactic bacteria was developed using the Neighbor-Joining method. It demonstrated that the *Lactobacillus sakei* and *Pediococcus pentosaceus* putative enzymes originated from the same ancestral node, which shares a common ancestor with Agdc1p, whereas *Enterococcus faecium, Streptococcus gallolyticus, Oenococcus oeni, Lactobacillus plantarum, Lactobacillus pentosus* and *Lactobacillus brevis* form other branches with common ancestors (Figure 1B).

### Generation of an Agdc1-6hp producing yeast strain

**Figure 2:**
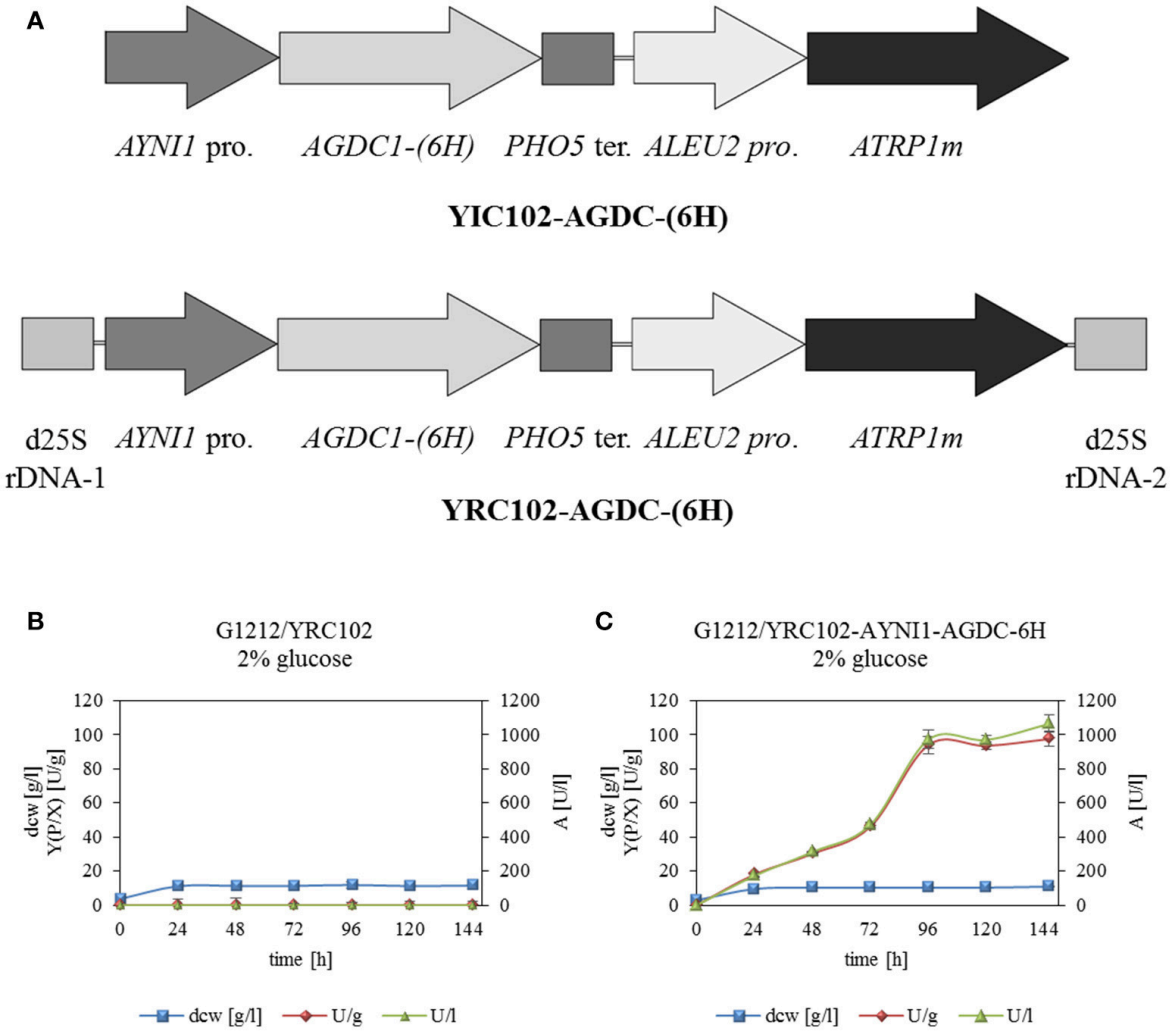
**(A)** Physical maps of YRC102-AYNI1-AGDC1-6H, YIC102-AYNI1-AGDC1-6H transformed into *A. adeninivorans* G1212. Both cassettes contained the selection marker module with *ATRP1m* gene fused to the *ALEU2* promoter and the expression module with *AYNI1* promoter—*AGDC1*-*6H* gene—*PHO5* terminator. In addition, YRC (*Asc*I fragment) is flanked by 25S rDNA sequences for targeting the cassette into genomic 25S rDNA. YIC (*Sbf* I fragment) contain the selection marker and expression module and was integrated randomly into the genome. **(B)** Time-course traces of *A. adeninivorans* G1212/YRC102 and **(C)** G1212/YRC102-AYNI1-AGDC1-6H grown on YMM-glucose-NaNO_3_ in shake flasks at 30°C for 146 h. Intracellular Agdc1-6hp activity (triangles) detected with gallic acid as substrate, calculated yield [Y(P/X)] (circles) and dry cell weight (squares). Measurements were done in triplicate.

*Arxula adeninivorans* G1212 [Δ*trp1*] served as a host strain for overexpression of *AGDC1* gene with a HisTag encoding sequence fused to the 3′-end of the ORF under control of the nitrate inducible *AYNI1* promoter (*A. adeninivorans* nitrate reductase promoter). Cassettes with the AGDC expression module (YRC102-AYNI1-AGDC1-6H, YIC102-AYNI1-AGDC1-6H-Figure 2A) and control (YRC102) were prepared as described in “Material and Methods.” After genome integration of the cassettes, a number of selected clones (YICs and YRCs) were passaged to establish high plasmid stability. The transformants were then cultivated in YMM-glucose-NaNO_3_ at 30°C for 48 h. The yeast cells were harvested, disrupted and screened for gallic acid decarboxylase activity. This activity was detected in all stabilized *A. adeninivorans* G1212/YRC102-AYNI1-AGDC1-6H and G1212/YIC102-AYNI1-AGDC1-6H strains. However transgenic yeast strains with genome integrated YRCs achieved approximately 2.2-fold higher enzyme activity than YIC transformants. In contrast, gallic acid decarboxylase activity was not detectable in the control strain *A. adeninivorans* G1212/YRC102 using glucose as carbon source. *A. adeninivorans* G1212/YRC102-AYNI1-AGDC1-6H with the highest gallic acid decarboxylase activity was used in further investigations.

AGDC1 expression and control strains *A. adeninivorans* G1212/YRC102-AYNI1-AGDC1-6H and G1212/YRC102 were cultivated in YMM-glucose-NaNO_3_ at 30°C and 180 rpm for 120 h and dry cell weight (dcw), gallic acid decarboxylase activity with gallic acid as substrate and yield [Y(P/X)] were determined each 24 h. The experiment was performed in triplicate.

All yeast strains reached a maximum dcw after 24 h of cultivation and then remained constant until the end of the experiment. In contrast gallic acid decarboxylase activity was detected in *A. adeninivorans* G1212/YRC102-AYNI1-AGDC1-6H only. This strain achieved its maximum enzyme activity of 972 U/l with 94 U/g dcw after 96 h (Figure 2B).

### Phenotype of transgenic *A. adeninivorans* strains

**Figure 3:**
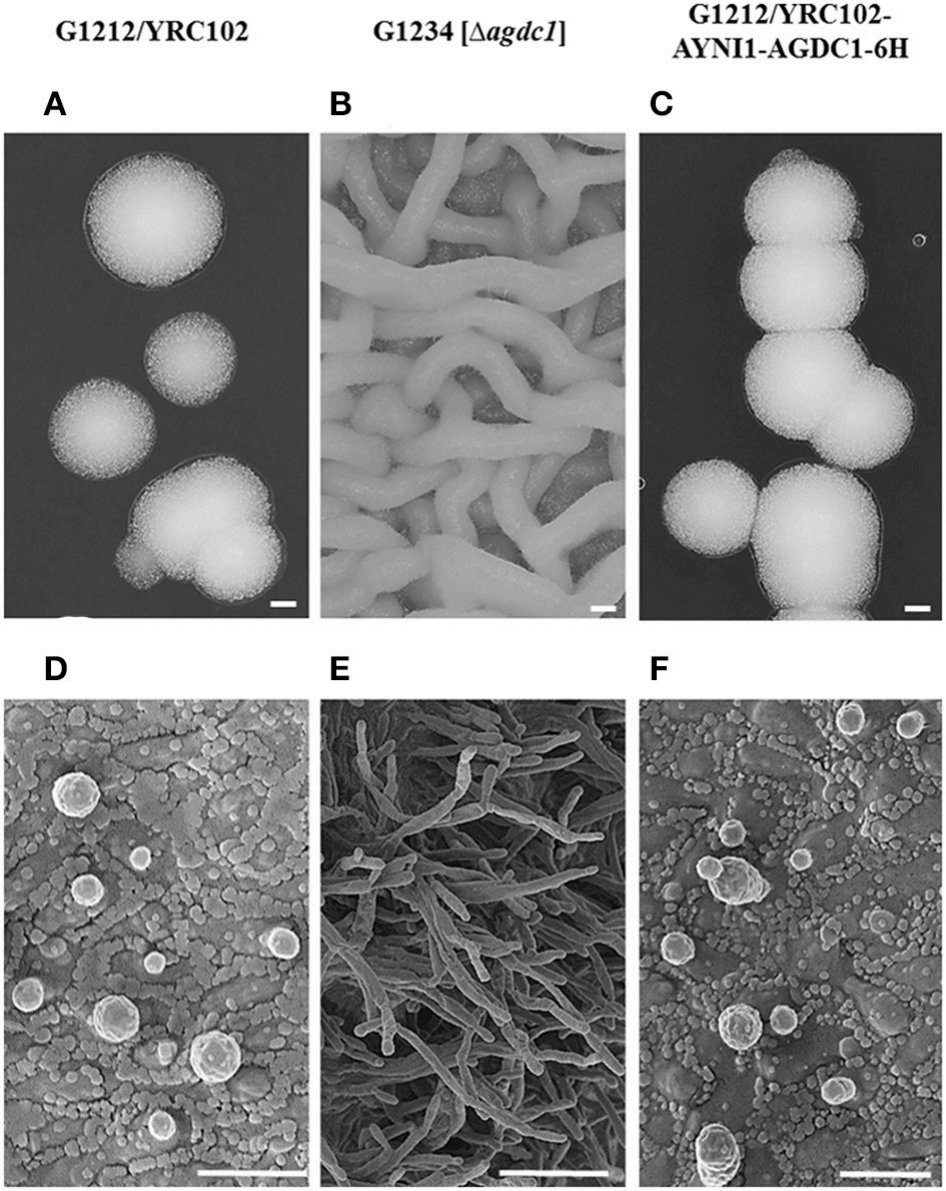
Microscopic analysis of *A. adeninivorans* G1212/YRC102, G1212/YRC102-AYNI1-AGDC1-6H and G1234 [Δ*agdc1*] cultivated on agar plates containing YMM-glucose-NaNO_3_ at 30°C for 48 h using a VHX-5000 Digital Microscope (Scale bar: **A–C**-200 μm) and a SEM FESEM S 4100 device (Scale bar: **D**-3 μm, **E**-9 μm, **F**-4 μm).

The deletion of gallic acid decarboxylase gene results in change of cells shape and through this, changes in morphology of colonies. The difference was visible already on the agar plate (YMM-NaNO_3_ supplemented with 2% glucose as sole source of carbon) without magnification. The microscopic analysis showed that the deletion mutant strains were growing as mycelia at 30°C; this phenomenon was not observed in the case of G1212/YRC102 control strain and G1212/YRC102-AYNI1-AGDC1-6H overexpression strain (Figure 3).

### Purification and characterization of Agdc1-6hp

*Arxula adeninivorans* G1212/YRC102-AYNI1-AGDC1-6H with HisTag encoding sequence at the 3′-end of the *AGDC1* ORF was selected for synthesis of recombinant Agdc1-6hp. Purification was by Immobilized Metal Affinity Chromatography (IMAC). Crude extract and a fraction containing purified Agdc1-6hp were analyzed on Coomassie-stained SDS-PAGE and Western blot.

**Figure 4:**
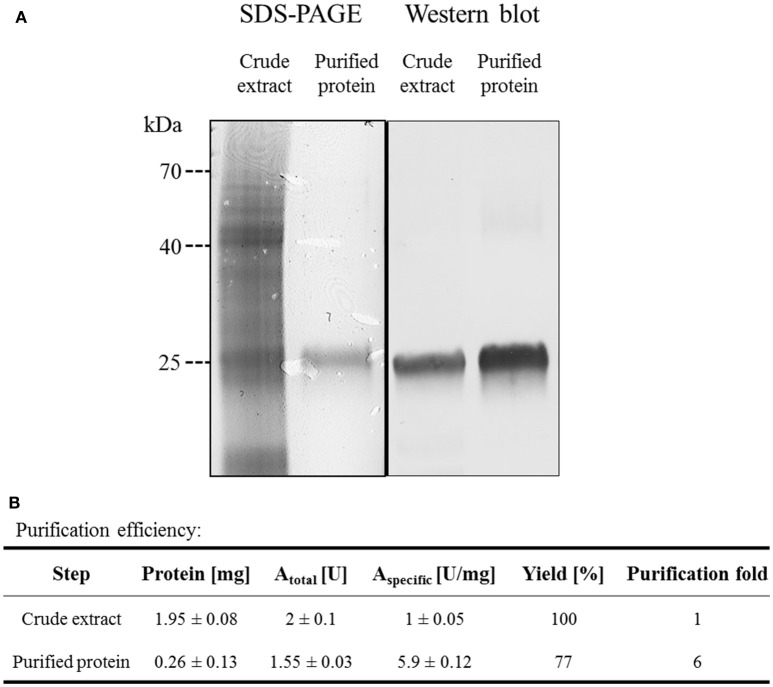
Purification of Agdc1-6hp on Ni Sepharose. **(A)** Crude extract and purified Agdc1-6hp visualized on Coomassie-stained SDS-PAGE (12%) and Western blot, using primary antibodies anti-polyhistidine from rabbit and secondary antibodies anti-rabbit from goat. **(B)** Summary of the Agdc1-6hp purification.

Predicted molecular mass of Agdc1-6hp is 27.3 kDa. The denatured protein was visible at around the 25 kDa position on SDS-PAGE and Western blot in both the crude extract and the purified protein fraction (Figure 4A). Gallic acid decarboxylase activity was measured for crude extract and purified Agdc1-6hp. Total activity in crude extract was 1 U/mg whereas it was 5.9 U/mg in the purified fraction, corresponding to 77% of yield. The specific activity was 1 and 5.9 U/mg in crude extract and the purified Agdc1-6hp fraction respectively (Figure 4).

The indicative molecular mass of native Agdc1-6hp was estimated by gel filtration on a Superdex™ 200 column (as described in “Material and methods”). The calculated molecular mass of the native enzyme was 27.1 kDa, whereas predicted molecular mass of the His-tagged Agdc1-6hp was 28.1 kDa. This data indicates that Agdc1-6hp is a monomeric protein.

*In vitro* study of Agdc1-6hp established the optimal conditions for enzymatic activity. The reaction mixture contained 0.5 mM gallic acid as substrate. The optimum pH was determined using the following buffers: 50 mM citrate-phosphate (pH 2.6–8), 50 mM sodium acetate (pH 3–6), 50 mM sodium citrate (pH 3.5–6.5), 50 mM potassium phosphate buffer (pH 5.8–6.8), 50 mM sodium phosphate (pH 5.5–8), and 50 mM TRIS-HCl (pH 7–9). Agdc1-6hp was most active when reaction was carried out in 50 mM potassium phosphate buffer pH 6.2 at 40°C. Enzyme activity of 80% was observed between pH 5.6–7.1 and at temperatures between 25 and 45°C. Some buffer systems reduced the activity of Agdc1-6hp, for example citrate-phosphate buffer exhibited 52% activity whereas sodium-citrate buffer had only 15% of activity in comparison to potassium phosphate buffer when the reaction was carried out under optimal conditions.

A study of thermostability demonstrated that Agdc1-6hp is stable between 4 and 30°C. After 24 h 100% of the initial activity was detected, however incubation at 35°C resulted in loss of 60% of initial activity after 24 h. Incubation at optimum temperature (40°C) resulted 100% gallic acid decarboxylase activity after only 7 h of incubation, however activity decreased drastically thereafter. Temperatures above 40°C led to a complete loss of activity after 4 h. Enzyme stored at −80°C exhibited no loss of activity even after 1 month of storage in these conditions.

A number of metal ions and reagents were tested to determine which acted as inhibitors and/or cofactors. When 1 mM Ni^2+^, Fe^2+^, Fe^3+^, Co^2+^, or Cu^2+^ were present in a reaction mixture, the enzyme was almost completely inhibited (relative activity <30%). In contrast addition of 1 mM Mg^2+^, Mn^2+^, Ca^2+^ K^+^, Na_2_S_2_O_3_, PEG4000, PEG6000, or PEG8000 to the reaction mixture had no significant influence on gallic acid decarboxylase activity. In all cases activities were >80% of activity in control reaction mixture. The slight positive effect was detected when 1 mM EDTA, ascorbic acid or DTT were present in reaction mixture (Table 2).

**Table 2:** Relative Agdc1-6hp activity [%] assayed for 0.5 mM gallic acid as substrate and presence of different additives in the reaction mixture.

**1 [mM]**	**Relative activity [%]**	**1 [mM]**	**Relative activity [%]**	**1 [mM]**	**Relative activity [%]**
Control (H_2_O)	100	ZnSO_4_	52	Na_2_S_2_O_3_	85
NiSO_4_	0	ZnCl_2_	60	PEG4000	91
NiCl_2_	0	AlCl_3_	73	PEG6000	95
FeSO_4_	0	MgSO_4_	84	PEG8000	95
FeCl_3_	24	MgCl_2_	84	Ascorbic acid	107
CoSO_4_	4	MnSO_4_	86	DTT	116
CoCl_2_	27	MnCl_2_	94	EDTA	146
CuSO_4_	4	CaSO_4_	84		
CuCl_2_	39	CaCl_2_	97		

The kinetic parameters of purified Agdc1-6hp were determined photometrically for gallic acid and protocatechuic acid. The K_m_ of Agdc1-6hp for gallic acid is 4 times lower than for protocatechuic acid (0.7 ± 0.2 mM and 3.2 ± 0.2 mM respectively). Turnover, k_cat_, is 42.0 ± 8.2 s^−1^ for gallic acid and 44.0 ± 3.4 s^−1^ for protocatechuic acid. However, the catalytic efficiency is much lower for protocatechuic acid (14.0 ± 2.0 [mM^−1^ s^−1^]) than for gallic acid (57.8 ± 7.6 [mM^−1^ s^−1^]) (Supplementary Table S1).

Five additional hydroxylated aromatic acids (3-hydroxybenzoic acid, 4-hydroxybenzoic acid, 2,3-dihydroxybenzoic acid, 2,4-dihydroxybenzoic acid, 2,5-dihydroxybenzoic acid) with different numbers and positions of hydroxyl groups were tested to determine the *in vitro* substrate specificity of Agdc1-6h. The enzyme, incubated in optimal conditions for 35 min, did not catalyze the decarboxylation of these compounds.

### Expression analysis of *AGDC1* on various carbon sources

**Figure 5:**
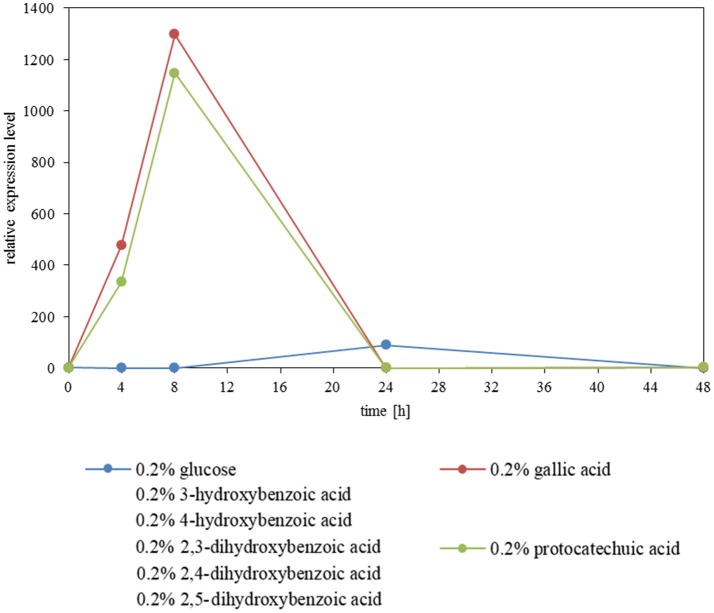
Influence of carbon sources on the gene expression level of *AGDC1* in *A. adeninivorans* G1212/YRC102 cultivated on YMM-NaNO_3_ supplemented with each 0.2% glucose, gallic acid, protocatechuic acid, 3-hydroxybenzoic acid, 4-hydroxybenzoic acid, 2,3-dihydroxybenzoic acid, 2,4-dihydroxybenzoic acid and 2,5-dihydroxybenzoic acid. Expression analysis was performed by nested quantitative real time PCR.

The expression level of *AGDC1* relative to the housekeeping genes *TFCI* and *ALG9* was analyzed by nested quantitative RT-PCR. *A. adeninivorans* G1212/YRC102 was cultivated on YMM-NaNO_3_ supplemented with 0.2% (w/v) of glucose or different hydroxylated aromatic acids as sole source of carbon. Samples were collected at different times and total RNA was isolated. An increase of gene transcription of *AGDC1* was observed when cells were cultivated on gallic acid and protocatechuic acid. None of the other carbon sources (glucose, 3-hydroxybenzoic acid, 4-hydroxybenzoic acid, 2,4-dihydroxybenzoic acid, 2,3-dihydroxybenzoic acid, 2,5-dihydroxybenzoic acid) induced the expression of *AGDC1*. The highest expression level was detected after 8 h of cultivation (Figure 5).

**Figure 6:**
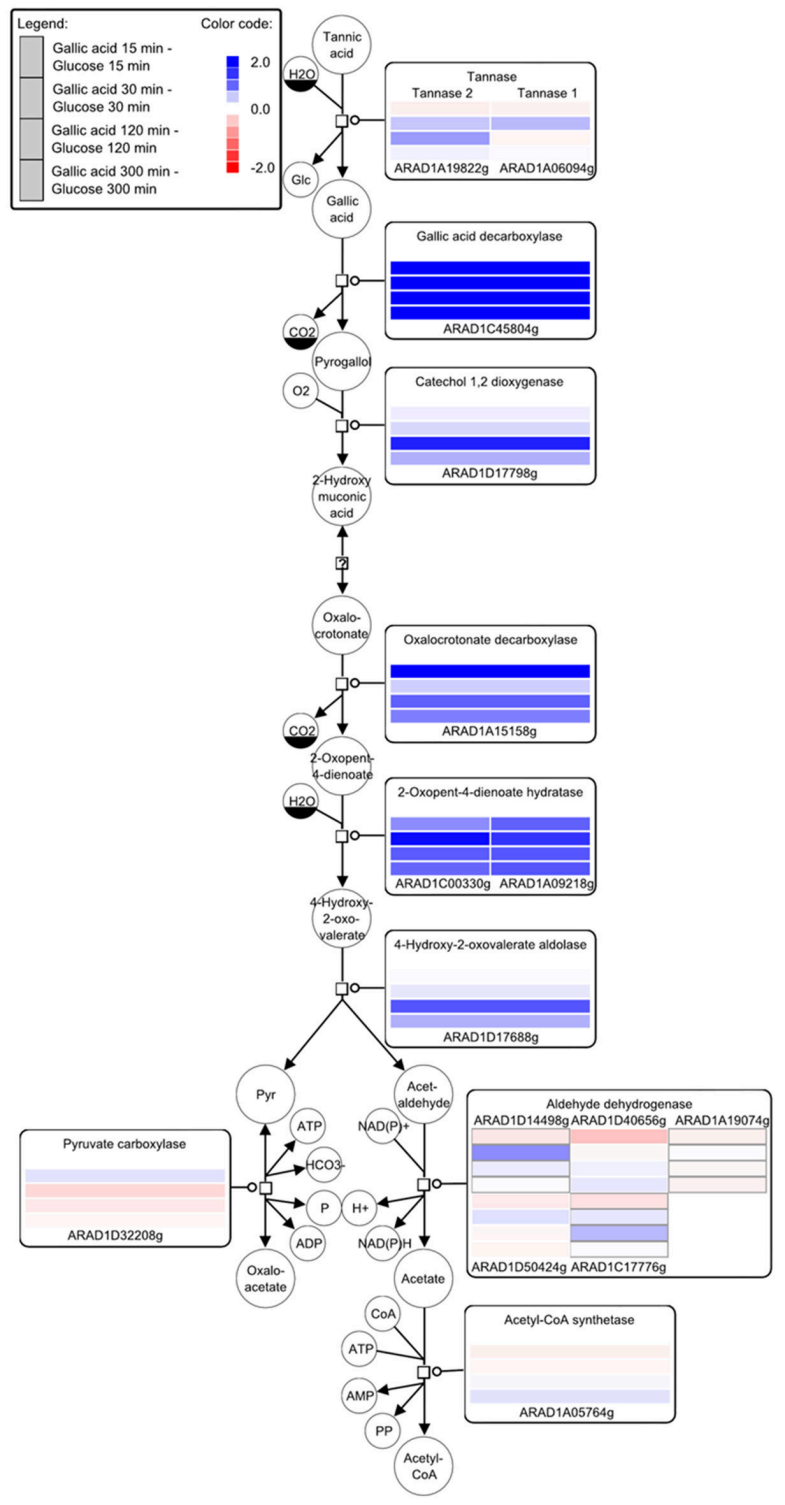
Key compounds of the tannin catabolism - microarray studies. The SBGN style metabolic network depicts reactions catalyzed by the corresponding enzymes (rectangular square). Enzymes are enriched with color-coded fold change values of time resolved expression data of the respective genes. The colors represent upregulation (blue) and downregulation (red) of genes in cells shifted to a medium containing gallic acid as the carbon source compared to cells grown with glucose. Metabolites or enzymes occurring multiple times in the metabolic network are decorated with a clone marker (e.g., NAD^+^) [produced using VANTED–(Junker et al., 2012; Rohn et al., 2012)].

In order to get information on whole transcriptome variations upon incubation of *A. adeninivorans* with gallic acid, microarray expression analysis was performed. The changes in genes expression of wildtype strain, *A. adeninivorans* LS3, cultivated in YMM-NaNO_3_ supplemented with 1% glucose and YMM-NaNO_3_ supplemented with 1% glucose plus 0.5% gallic acid were analyzed. Cells were harvested after 15 min, 30 min, 5 h, and 12.5 h of incubation in shaking flasks at 30°C and 180 rpm. Total RNA was isolated and analysis of microarray data allowed recognition of the candidate genes involved in tannic acid degradation pathway (Figure 6). Significant upregulation was observed for *gallic acid decarboxylase 1* (*AGDC1*) and *tannase 1* (*ATAN1*) genes, as well as for genes annotated as putative *tannase 2* (*ATAN2*), *catechol-1,2-dioxygenase, oxalocrotonate decarboxylase, 2-oxopent-4-dienoate hydratase, 4-hydroxy-2-oxovalerate aldolase*. These data suggest that additional enzymes are involved in tannic acid degradation. These enzymes convert the pyrogallol into pyruvate and acetaldehyde and make them available to the central metabolism (Figure 6). In addition, microarray data showed the upregulation of genes involved in β-oxidation process but not in the glyoxylate cycle, methyl citrate cycle and in the catabolism of the branched-chain amino acids valine, leucine and isoleucine (Supplementary Figures S1–S4).

### Gallic acid tolerance by *A. adeninivorans*

The growth behavior of *A. adeninivorans* G1212/YRC102 on different gallic acid concentrations was investigated. The cells were cultivated at 30°C, 180 rpm on YMM-NaNO_3_ supplemented with 0.05–2% (w/v) gallic acid as sole source of carbon. Growth occurred in all concentrations and the growth profile at all concentrations was characterized by long adaptation phase. Cell growth required 48 h of cultivation (Supplementary Figure S5) to start to increase in rate. The OD_600 nm_ values were strongly correlated to the amount of carbon sources in medium. Despite the long adaptation phase, no growth inhibition was observed. Additionally, the final optical densities, reached during cultivation on gallic acid were lower than those seen in growth on medium supplemented with an equal concentration of glucose.

### Role of Agdc1p in *A. adeninivorans* on metabolizing gallic acid

**Figure 7:**
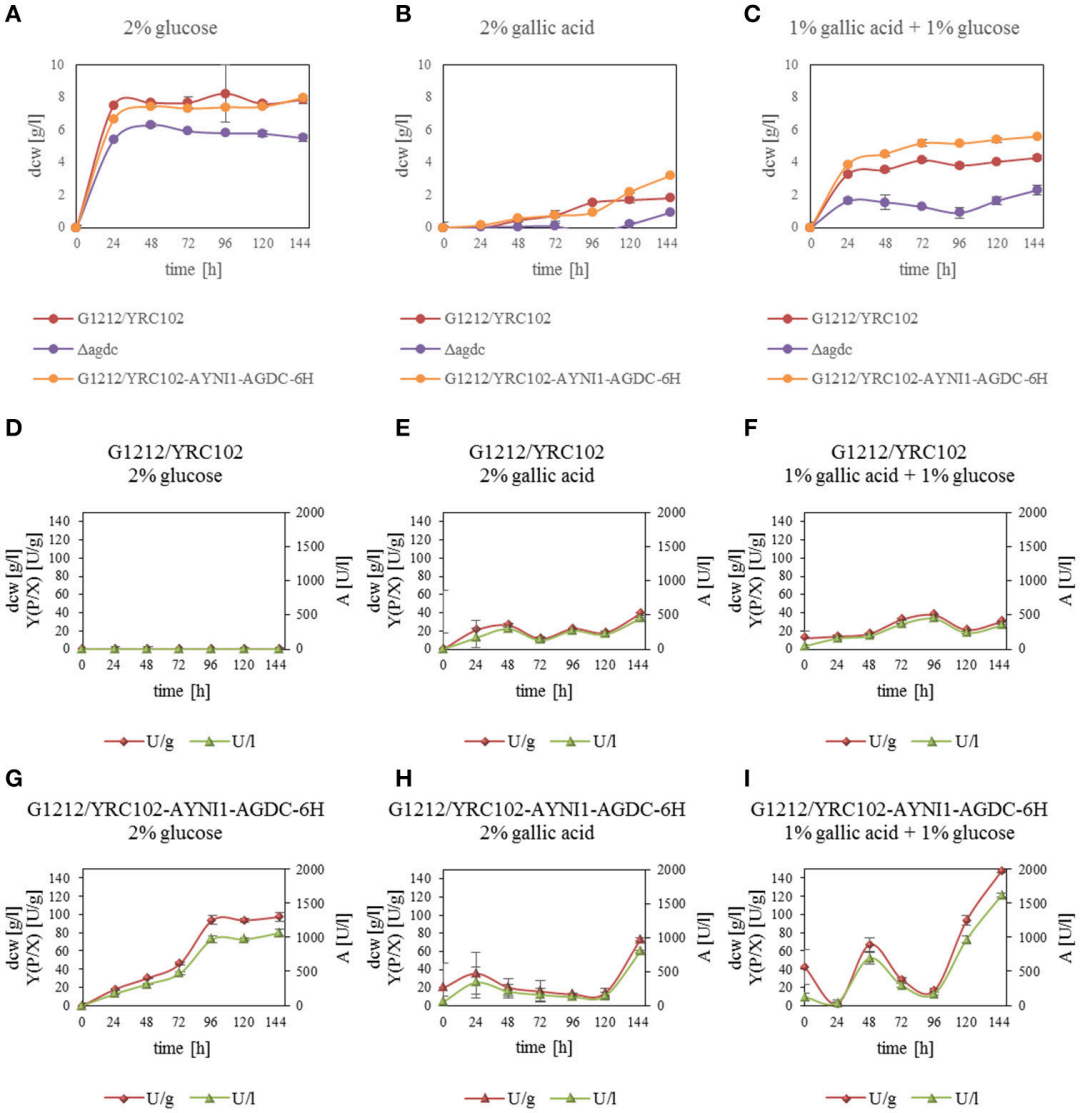
**(A–C)** Influence of carbon source on the dry cell weight of *A. adeninivorans* G1212/YRC102, G1212/YRC102-AYNI1-AGDC1-6H and G1234 [Δ*agdc1*] as well as **(D–I)** Agdc1-6hp activity and yield [Y(P/X)] with cultivation in YMM-NaNO_3_ with 2% glucose, 2% gallic acid or 1% glucose plus 1% gallic acid as carbon sources.

**Figure 8:**
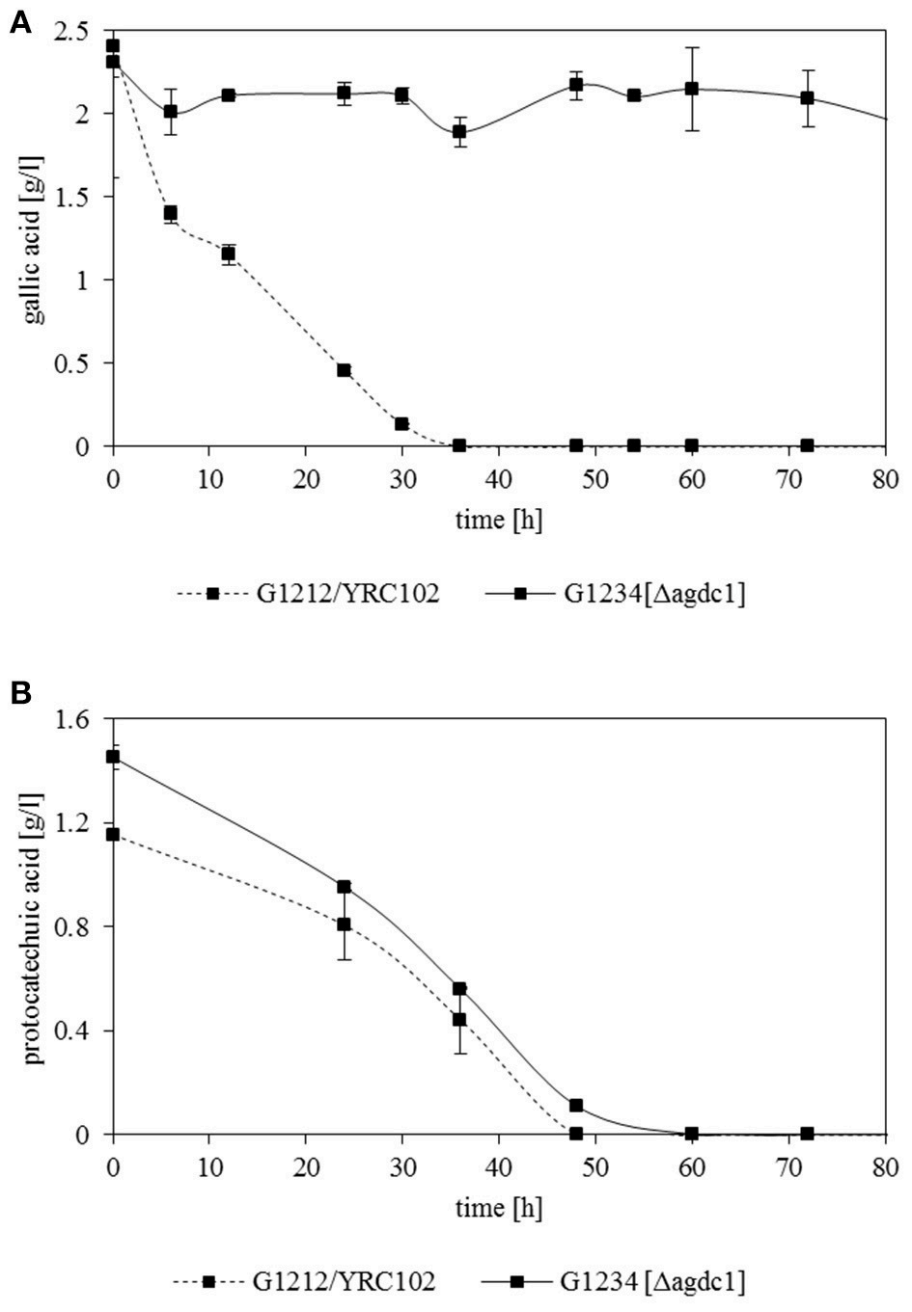
Degradation of **(A)** gallic acid and **(B)** protocatechuic acid by *A. adeninivorans* G1212/YRC102 and G1234 [Δ*agdc1*] cultured in YMM-NaNO_3_ with 0.25% (w/v) gallic acid or protocatechuic acid as sole carbon source. The metabolites accumulated in the supernatant were analyzed by GC-MS.

Previous investigations indicated that Agdc1p is an enzyme expressed by a gallic acid inducible gene. To analyse its *in vivo* role, three *A. adeninivorans* strains G1212/YRC102, G1212/YRC102-AYNI1-AGDC1-6H, and G1234 [Δ*agdc1*] were studied. All strains were cultivated at 30°C up to 146 h on YMM-NaNO_3_ supplemented with 2% (w/v) glucose, 2% (w/v) gallic acid, or 1% (w/v) gallic acid plus 1% (w/v) glucose. Dcw, gallic acid decarboxylase activity and its yield [Y(P/X)] were determined. Deletion of *AGDC1* contributed to cells death or growth inhibition when gallic acid was present in culture medium (Figure 7). No gallic acid decarboxylase activity was detected in crude extract of *A. adeninivorans* G1234 [Δ*agdc1*] in all cultivation conditions. Additional metabolite analysis showed that gallic acid remains in the culture medium during the entire cultivation. This was not detected when *A. adeninivorans* G1234 [Δ*agdc1*] was cultivated in a medium with protocatechuic acid as the carbon source (Figure 8). In contrast gallic acid decarboxylase was synthesized and accumulated in the control strain *A. adeninivorans* G1212/YRC102 when gallic acid was present in the medium. On the other hand, the overexpression strain *A. adeninivorans* G1212/YRC102-AYNI1-AGDC1-6H exhibited activity during cultivation on glucose and reached 1,064 U/l and 97.5 U/g between 96–146 h of cultivation. The highest enzyme activity of 1,617 U/l and 148 U/g was detected in the crude extract of *A. adeninivorans* G1212/YRC102-AYNI1-AGDC1-6H after cultivation on a mixture of gallic acid and glucose. In comparison, the cultivation of G1212/YRC102 in the same conditions exhibited maximal 446.7 U/l and 37.5 U/g. However, the overexpression of *AGDC1* did not significantly improve the cells growth on gallic acid (Figure 7).

**Figure 9:**
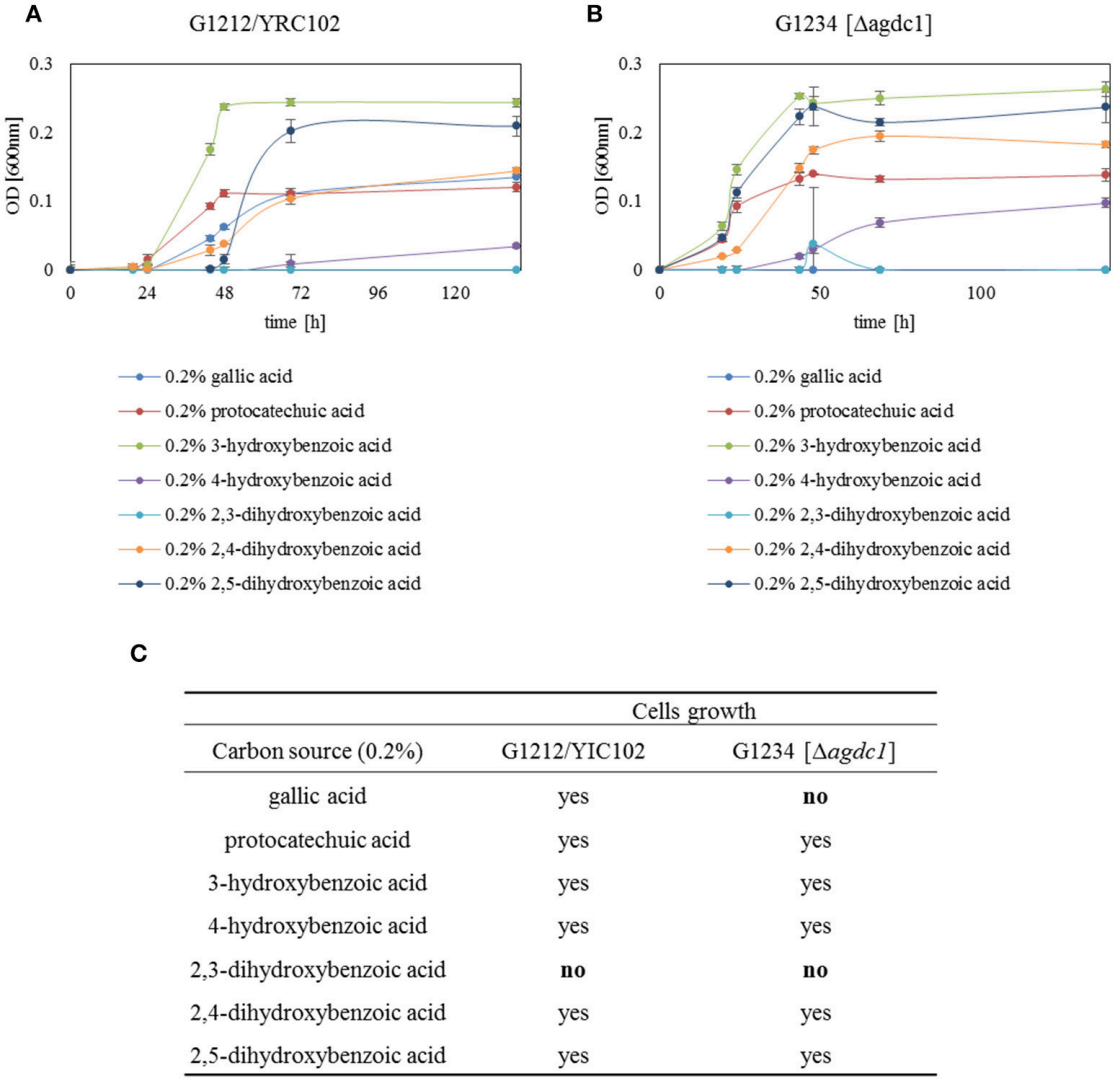
Growth behavior of **(A)** *A. adeninivorans* G1212/YRC102 and **(B)** G1234 [Δ*agdc1*] on YMM-NaNO_3_ with different hydroxylated aromatic acids (each 0.2% gallic acid, protocatechuic acid, 3-hydroxybenzoic acid, 4-hydroxybenzoic acid, 2,3-dihydroxybenzoic acid, 2,4-dihydroxybenzoic acid and 2,5-dihydroxybenzoic acid) as carbon source. Measurements were made in triplicate. **(C)** Summarize of growth behavior of *A. adeninivorans* on different hydroxylated aromatic acids as sole source of carbon.

To demonstrate that the main role of gallic acid decarboxylase in *A. adeninivorans* is to degrade gallic acid, *A. adeninivorans* G1212/YRC102 and G1234 [Δ*agdc1*] were cultivated for 48 h on YMM-NaNO_3_ supplemented with 0.2% (w/v) of different hydroxylated aromatic acids as sole source of carbon. Samples were collected at 0, 30, and 48 h of cultivation for metabolite analysis. This investigation demonstrated that *A. adeninivorans* G1212/YRC102 is unable to grow on 2.3-dihydroxybenzoic acid. However, this strain was able to grow on all other carbon sources (gallic acid, protocatechuic acid, 3-hydroxybenzoic acid, 4-hydroxybenzoic acid, 2,4-dihydroxybenzoic acid and 2,5-dihydroxybenzoic acid) with different adaptation times. The deletion mutant was unable to grow on gallic acid and 2.3-dihydroxybenzoic acid as carbon source. In all other cases, the adaptation phase of *A. adeninivorans* G1234 [Δ*agdc1*] was shorter and cells grew faster compared to the control strain. Interestingly, the best growth of both strains was observed for 3-hydroxybenzoic acid and the slowest growth was with 4-hydroxybenzoic acid (Figure 9).

**Figure 10:**
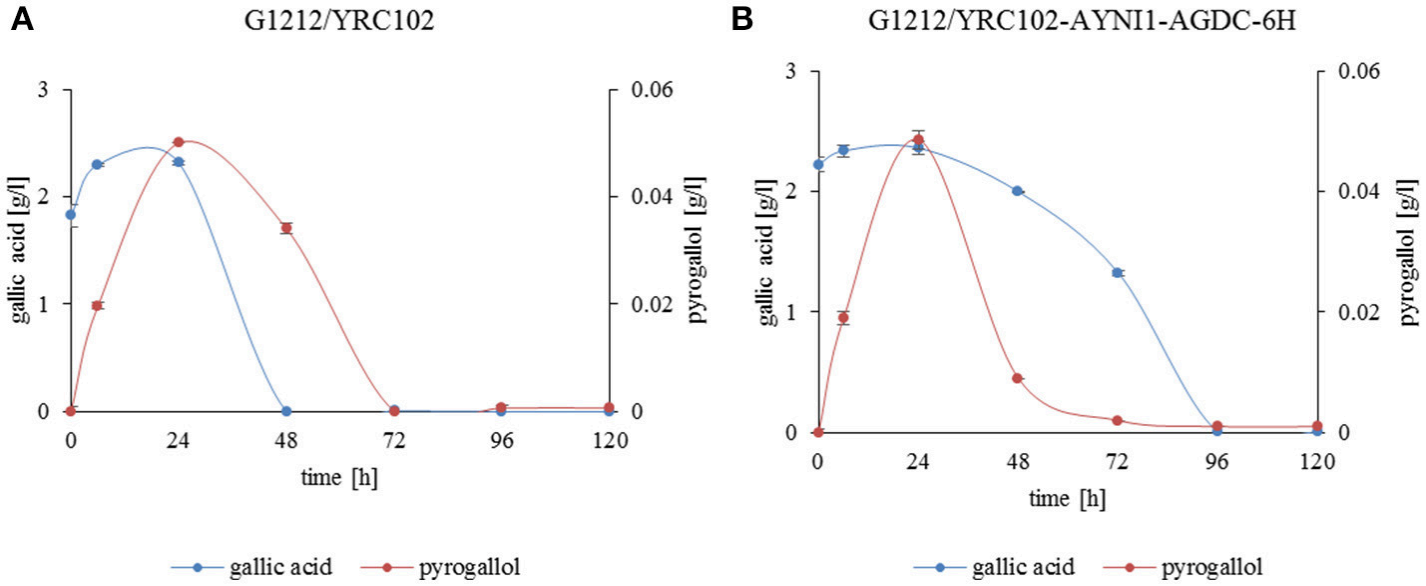
Gallic acid degradation (blue line) and pyrogallol production (red line) by **(A)** *A. adeninivorans* G1212/YRC102 and **(B)** G1212/YRC102-AYNI1-AGDC1-6H cultivated on YMM-NaNO_3_ with 0.25% gallic acid plus 0.5% glucose.

*In vivo* transformation of gallic acid to pyrogallol by *A. adeninivorans* was investigated. *A. adeninivorans* G1212/YRC102 and G1212/YRC102-AYNI1-AGDC1-6H were cultivated on YMM-NaNO_3_ supplemented with 0.25% gallic acid plus 0.5% glucose as the carbon source. The metabolites were analyzed by GS-MS. Complete consumption of gallic acid occurred after 48 h cultivation of the control strain and after 96 h of the G1212/YRC102-AYNI1-AGDC1-6H strain. The maximum extracellular pyrogallol production was detected after 24 h incubation. The yield, calculated as the % of substrate converted into the product detected in the culture medium, was similar for both strains; approximately 2.7% for control strain and 2.2% for overexpression strain (Figure 10). Pyrogallol was only product detected during degradation of gallic acid by *A. adeninivorans*.

**Figure 11:**
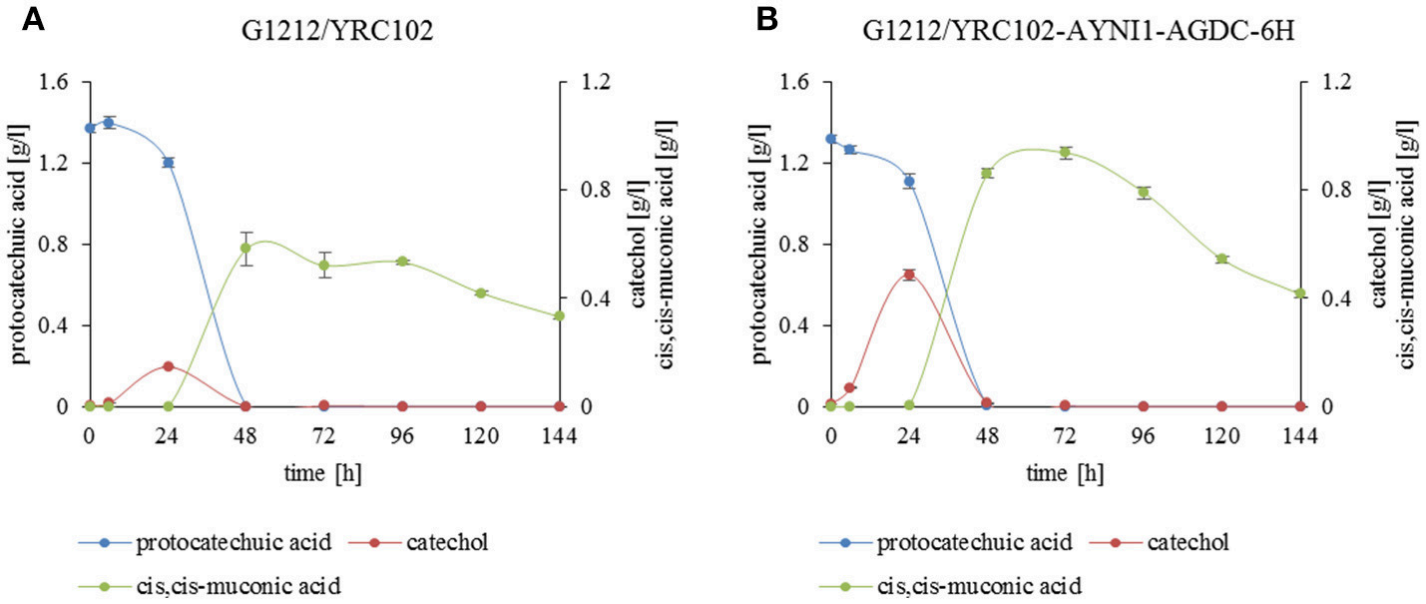
Substrate and product accumulation during the degradation of protocatechuic acid by *A. adeninivorans* **(A)** G1212/YRC102 and **(B)** G1212/YRC102-AYNI1-AGDC1-6H. Cells were cultivated on YMM-NaNO_3_ with 0.25% protocatechuic acid plus 0.5% glucose. Compounds present in culture medium were detected via GC-MS.

Degradation of protocatechuic acid in *A. adeninivorans* was also studied and the products produced during the degradation of protocatechuic acid by G1212/YRC102 and G1212/YRC102-AYNI1-AGDC1-6H were analyzed. The products detected in the culture medium during cultivation were catechol, *cis,cis*-muconic acid and trace amounts of 3-hydroxybenzoic acid, 4-hydroxybenzoic acid, 1,4-dihydroxybenzene and 1,3,4-benzentriol (Figures 11A,B).

In addition, a time course analysis of protocatechuic acid consumption (Figures 11A,B) by the overexpression strain *A. adeninivorans* G1212/YRC102-AYNI1-AGDC1-6H was conducted. The protocatechuic acid was completely degraded after 48 h and the maximum catechol concentration was detected after 24 h of cultivation. *Cis,cis*-muconic acid appeared even later, and the highest concentration was observed at 48 h of cultivation of G1212/YRC102 strain and after 72 h in G1212/YRC102-AYNI1-AGDC1-6H. The yield, calculated as the % of protocatechuic acid converted into the product (*cis,cis*-muconic acid) detected in the culture medium, was 42.5% for G1212/YRC102 and 71.2% for G1212/YRC102-AYNI1-AGDC1-6H (Figure 11B).

## Discussion

Decarboxylation of hydroxylated aromatic acids has been reported in bacteria and fungi. A well-known example is the non-oxidative decarboxylation of gallic acid, which leads to the synthesis of pyrogallol (Grant and Patel, 1969; Yoshida et al., 1982; Li and Wang, 2015). The yeast *A. adeninivorans* LS3 has been described as a microorganism able to utilize some hydroxylated aromatic acids such as tannic acid, gallic acid, protocatechuic acid, as a sole source of carbon and energy (Sietmann et al., 2010). The authors described the pathways for the degradation of gallic acid, protocatechuic acid and 4-hydroxybenzoic acid in the LS3 wildtype strain. However, the enzymes involved in the degradation of these compounds have not been identified and characterized. The complete genome data of *A. adeninivorans* has been available since 2014 (Kunze et al., 2014), which has allowed detailed studies of the tannic acid degradation pathway.

For the first time, it has been demonstrated that deletion of the gene encoding gallic acid decarboxylase causes changes in the morphology of *A. adeninivorans* cells. This organism is a dimorphic microorganism but the exact physiological regulation of dimorphism is still unknown. The only factor known to initiate a change of cell shape from budding cells to mycelia is a temperature above 42°C. It is not clear why the lack of this enzyme causes the change in morphology and it is not known if deletion of further enzymes involved in this pathway will have a similar effect. Gallic acid decarboxylase plays very important role in the metabolism of tannins and phenol derivatives, which are toxic for cells and the lack of this enzyme may cause cell stress and possible changes in shape. It is also possible that the enzyme is somehow connected with a signaling pathway which is controlled by temperature allowing mycelia to form at 30°C. Finally, it is possible that the enzyme plays some other important roles which are still not known and further study is required to explain the enzymes role in the dimorphism of *A. adeninivorans*.

Gallic acid decarboxylase is one enzyme of this pathway that transforms gallic acid to pyrogallol. Gallic acid decarboxylases described so far are known for their instability due to a high oxygen sensitivity, and only a few have been successful purified (Zeida et al., 1998; Jiménez et al., 2013). Some gallic acid decarboxylases have been partially characterized, whereas the rest of them remain uncharacterized due to their complete loss of activity after purification (Grant and Patel, 1969; Brune and Schink, 1992; Yoshida et al., 2010). In this study gallic acid decarboxylase from *A. adeninivorans* fused with C-terminal HisTag region (Agdc1-6hp) has been successfully purified and characterized for the first time. Selection of the appropriate buffer and pH seems to be crucial for the storage of purified Agdc1-6hp. The enzyme stored in crude extract at 4°C for 5 days led to 42% loss of activity which is similar to the results obtained by Zeida et al. (1998). In 50 mM potassium phosphate buffer (pH 6) the purified enzyme retained 60% activity after 9 days at 4°C. A decrease in enzyme activity was not observed after one-month storage at −80°C. This suggests the possibility for long-term storage of Agdc1-6hp. The native molecular mass is approximately 27 kDa which indicates a monomeric enzyme. This is uncommon in comparison to known bacterial gallic acid decarboxylases, some of which consist of six subunits (Zeida et al., 1998; Jiménez et al., 2013) and decarboxylases from *Bacillus megaterium* and *Clostridium hydroxybenzoicum* possess a minimum two enzyme subunits (He and Wiegel, 1995, 1996; Omura et al., 1998).

Several metal ions were tested as potential cofactors for Agdc1p. Almost all metal ions had negative influence and some like Fe^2+^ or Ni^2+^ completely inhibited the activity. Na_2_S_2_O_3_ used as stabilizer for gallic acid decarboxylases from *Pantotea agglomerans* T71 or *Lactobacillus plantarum* WCFS1 did not show any stabilization effect on Agdc1p. Instead, it resulted in decrease of activity up to 85% (Zeida et al., 1998; Jiménez et al., 2013). Addition of DTT and ascorbic acid to the reaction mixture exhibited 107 and 116% of relative activity respectively, compared to the control. Addition of EDTA alone led to 146% relative activity which is in contrast to the inhibitory effect of EDTA on gallic acid decarboxylase observed in *P. agglomerans* T71 (Zeida et al., 1998). Because the purification procedure was carried out on Ni Sepharose column, it is possible that the EDTA does not directly affect the enzyme but acts by chelating the nickel residues after purification, which could have inhibitory effects on the enzyme activity. However, it is also possible that EDTA positively affects the enzyme. *In vitro* investigation of kinetic and substrate specificity of Agdc1-6hp indicated that the enzyme has four times higher affinity for gallic acid than for protocatechuic acid. The K_m_ value for gallic acid is 0.7 ± 0.2 mM and 3.2 ± 0.2 mM for protocatechuic acid. In comparison, the K_m_ for gallic acid is 0.96 mM for gallic acid decarboxylase from *P. agglomerans* T71 which is unable to decarboxylate other gallic acid analogs (Zeida et al., 1998). The ability of Agdc1-6hp to decarboxylate other hydroxylated aromatic acids *in vitro* has not been confirmed. Most of the gallic acid decarboxylases, which have been characterized have a limited substrate spectrum (He and Wiegel, 1995, 1996; Zeida et al., 1998). The 3,4-dihydroxybenzoate decarboxylase from *Enterobacter cloacae* P can decarboxylate protocatechuic acid, but not gallic acid (Yoshida et al., 2010).

The cultivation of the control strain, *A. adeninivorans* G1212/YRC102, on YMM-glucose-NaNO_3_ did not reveal gallic acid decarboxylase activity during the cultivation. This suggests that Agdc1p is an enzyme encoded by an inducible gene. Nested qRT-PCR assays confirmed the inducibility of the *AGDC1* gene in presence of gallic acid and also protocatechuic acid. In contrast, glucose and the other selected hydroxybenzoic acid compounds were not able to induce the *AGDC1* gene. The most vigorous gallic acid degrading microorganisms contain inducible *GDC* genes (Yoshida and Yamada, 1985; O'Donovan and Brooker, 2001; Gauri et al., 2013; Jiménez et al., 2013; Li and Wang, 2015; Reverón et al., 2015). An exception is *Klebsiella aerogenes* where decarboxylation of gallic acid, protocatechuic acid and 2.5-dihydroxybenzoic acid were shown to be constitutive (Grant and Patel, 1969).

*A. adeninivorans* tolerates as much as 2% (w/v) gallic acid however, regardless of the gallic acid concentration, the growth curves exhibit long lag phases. Since gallic acid is a weak acid, its presence results in the lowering of the pH value of the culture medium a moderate amount e.g. supplementation with 1% (w/v) gallic acid lowered the pH value to approximately 3.8. At this pH, gallic acid is undissociated and can go across the yeast cell membrane. Inside the cell, the higher pH causes the gallic acid to dissociate, which reduces the pH_*i*_ to below the physiological range of the cell and thus interferes homeostasis. Both factors generate antimicrobial properties of hydroxylated aromatic acids, generating cell stress, extending the lag phase and inhibiting cell growth (Noda et al., 1982; Restaino et al., 1982; Beales, 2003; Ibraheem and Ndimba, 2013). An experiment which cultivated *A. adeninivorans* on a medium with a constant pH 6 (optimal for growth of *A. adeninivorans*) showed that cells were unable to degrade gallic acid resulting in cell death (data not showed). The overexpression of *AGDC1* did not shorten the lag phase or increase the cell growth period significantly over 120 h (Figure 7). Also, the activity of Agdc1p was lower and appeared later in comparison to cultivation on glucose. These results suggest a stress response at the level of gene expression generated by gallic acid. A longer log phase was also observed when *A. adeninivorans* was cultivated on other hydroxylated aromatic acids as sole source of carbon which indicates a similar effect on the cells (Figure 9).

The deletion of *AGDC1* in *A. adeninivorans* genome results in cell death when cells were cultivated on gallic acid (Figure 8). This suggests that there is no other pathway able to degrade gallic acid which is supported by extracellular metabolite analysis of the control strain, *A. adeninivorans* G1212/YRC102, which showed only the production of pyrogallol. In the previous study Sietmann et al. (2010) reported that in *A. adeninivorans*, oxidative and non-oxidative decarboxylation of protocatechuic acid occur simultaneously; indeed the G1234 [Δ*agdc1*] was still able to grow and degrade protocatechuic acid. Analysis of the metabolites in culture medium showed that control strain and the overexpression strain produced catechol and *cis,cis*-muconic acid. Trace amounts of 3-hydroxybenzoic acid, 4-hydroxybenzoic acid, 1,4-dihydroxybenzene and 1,3,4-benzentriol were also detected. Our investigations have confirmed that the degradation of protocatechuic acid involves gallic acid decarboxylase and other enzymes.

To confirm which compounds are produced during the cultivation of *A. adeninivorans* on protocatechuic acid, further investigations are required. Cultivation of *A. adeninivorans* G1234 [Δ*agdc1*] on media containing different hydroxylated aromatic acids as sole source of carbon revealed that only cultivation on gallic acid led to cell death (Figure 9). This indicates that gallic acid decarboxylase is the key enzyme in the degradation of gallic acid by *A. adeninivorans*. Furthermore, we determined that all of the tested aromatic acids except 2,3-dihydroxybenzoic acid can serve as sole source of carbon for *A. adeninivorans*. However, the first step of degradation is not carried out by gallic acid decarboxylase. Considering all aspects of growth and gene expression analysis, it seems that the tannic acid degradation pathway is used exclusively for tannins and gallic acid. Protocatechuic acid can be degraded also by this pathway, but the other aromatic acids are degraded through a separate pathway(s).

Interestingly the overexpression of *AGDC1* did not increase the accumulation of extracellular pyrogallol when cells were cultivated on glucose and gallic acid. The yield was approximately the same for both the control and the overexpression strain. The reason could be that most extracellular pyrogallol is accumulated in the first 24 h of cultivation. At this time, the cells are under strong stress conditions created by gallic acid. This results in low gene expression and low recombinant enzyme content. Probably the pre-adaptation of cells on low gallic acid concentration increases the yield of pyrogallol in the strain with overexpression of *AGDC1*. To obtain a higher yield of extracellular pyrogallol, further engineering of *A. adeninivorans* and optimization of the cultivation conditions are necessary.

Unexpectedly, the overexpression of gallic acid decarboxylase contributed to the accumulation of *cis,cis*-muconic acid. The yield increased almost by 71.2% over the control which suggests that *A. adeninivorans* could be an efficient synthesizer of this difficult to produce molecule. Nevertheless, further investigation and optimization are necessary to demonstrate this possibility.

The microarray data revealed the genes involved in the degradation of gallic acid. The *AGDC1* and *catechol-1,2-dioxyenase* genes encode the first two enzymes involved in the degradation of gallic acid which cleave the aromatic ring, resulting in the production of pyruvate and acetaldehyde. This is similar to the situation in *Eubacterium oxidoreducens* and *Pelobacter acidigallici* (Krumholz et al., 1987; Brune and Schink, 1992). The highest upregulation of genes in the presence of gallic acid are found in the ß-oxidation pathway. It is still an open question whether or not transporters are involved in the transport of gallic acid into *A. adeninivorans* cells. Microarray data did not exhibit any potential genes which could be recognized as gallic acid transporter(s) and there is little in the literature concerning the transport of hydroxylated aromatic acids in microorganisms. Two possibilities exist, either *A. adeninivorans* does not have transporters, and therefore gallic acid couldn't enter the cells at pH 6 because it is in the dissociated form or there exist pH dependent transporter(s), which are not yet annotated in the genome.

In this study, physiological and transcriptome analysis was used to understand the catabolism of hydroxylated aromatic acids in *A. adeninivorans*. We confirmed that the genes encoding enzymes involved catabolic pathway of gallic acid are induced and aromatic acid substrates are the inducers. The construction of gallic acid decarboxylase disruption mutants revealed that gallic acid decarboxylase Agdc1p is the only enzyme responsible for transformation of gallic acid. However, other enzymes were demonstrated to be responsible for the transformation of other hydroxylated aromatic acids.

Gallic acid decarboxylase affinity for gallic acid, its substrate specificity and the possibility of long-term storage of the Agdc1p will be advantageous if the enzyme were to be commercialized. The enzyme may have useful industrial applications e.g. in bioremediation processes or in chemical synthesis. Further culture optimization as well as strain engineering could also contribute to more efficient conversion of protocatechuic acid to *cis,cis*-muconic acid which may also have industrial application.

## Author contributions

AM and EB carried out the construction of the *E. coli* and *A. adeninivorans* strains, enzyme activities determination and Agdc1p analysis. AM prepared the quantitative reverse transcriptase PCR analysis. AM, AH, MM, US, and SW participated in microarray design, hybridization, gene annotation and gene expression analysis as well as visualization, AM and JR in GC–MS analysis. MM and AM prepared the microscopic analysis. RB and FS gave several useful suggestions. AM, KB, and GK drafted the manuscript. All authors read and approved the final manuscript.

### Conflict of interest statement

The authors declare that the research was conducted in the absence of any commercial or financial relationships that could be construed as a potential conflict of interest.

## Abbreviations

GDC: gallic acid decarboxylases
*AGDC* gene: *gallic acid decarboxylase 1* gene from *A. adeninivorans*
Agdc1p: gallic acid decarboxylase 1 protein from *A. adeninivorans*
dcw: dry cell weight
YRC: yeast rDNA integrative expression cassette
YIC: yeast integrative expression cassette
YEPD: complex medium
YMM: yeast minimal medium
U: unit
GA: gallic acid
PA: protocatechuic acid
3-HBA: 3-hydroxybenzoic acid
4-HBA: 4-hydroxybenzoic acid
2,3-DHBA: 2,3-dihydroxybenzoic acid
2,4-DHBA: 2,4-dihydroxybenzoic acid
2,5-DHBA: 2,5-dihydroxybenzoic acid
ORF: open reading frame.

## References

[B1] Badhani B. Sharma N. Kakkar R. (2015). Gallic acid: a versatile antioxidant with promising therapeutic and industrial applications. RSC Adv. 5, 27540–27557. 10.1039/C5RA01911G

[B2] Beales N. (2003). Adaptation of microorganisms to cold temperatures, weak acid preservatives, low pH, and osmotic stress: a review. Compr. Rev. Food Sci. Food Saf. 3, 1–20. 10.1111/j.1541-4337.2004.tb00057.x 33430556

[B3] Böer E. Bode R. Mock H. P. Piontek M. Kunze G. (2009a). Atan1p-an extracellular tannase from the dimorphic yeast *Arxula adeninivorans*: molecular cloning of the *ATAN1* gene and characterization of the recombinant enzyme. Yeast 26, 323–337. 10.1002/yea.1669 19387973

[B4] Böer E. Piontek M. Kunze G. (2009b). Xplor 2 - an optimized transformation/expression system for recombinant protein production in the yeast *Arxula adeninivorans*. Appl. Microbiol. Biotechnol. 84, 583–594. 10.1007/s00253-009-2167-5 19672589

[B5] Böer E. Schröter A. Bode R. Piontek M. Kunze G. (2009c). Characterization and expression analysis of a gene cluster for nitrate assimilation from the yeast *Arxula adeninivorans*. Yeast 26, 83–93. 10.1002/yea.1653 19191338

[B6] Bradford M. (1976). A rapid and sensitive method for the quantitation of microgram quantities of protein utilizing the principle of protein-dye binding. Anal. Biochem. 72, 248–254. 10.1016/0003-2697(76)90527-3 942051

[B7] Brune A. Schink B. (1992). Phloroglucinol pathway in the strictly anaerobic *Pelobacter acidigallici*: fermentation of trihydroxybenzenes to acetate via triacetic acid. Arch. Microbiol. 5, 157–417. 10.1007/BF00249098

[B8] Gauri S. S. Mandal S. M. Atta S. Dey S. Pati B. R. (2013). Novel route of tannic acid biotransformation and their effect on major biopolymer synthesis in *Azotobacter* sp. SSB81. J. Appl. Microbiol. 114, 84-95. 10.1111/jam.12030 23035941

[B9] Gienow U. Kunze G. Schauer F. Bode R. Hofemeister J. (1990). The yeast genus *Trichosporon* spec. LS3; molecular characterization of genomic complexity. Zbl. Mikrobiol. 145, 3–12. 2330765

[B10] Grant D. J. W. Patel J. C. (1969). The non-oxidative decarboxylation of *p*-hydroxybenzoic acid, gentisic acid, protocatechuic acid and gallic acid by *Klebsiella aerogenes (Aerobacter aerogenes)*. Antonie Van Leeuwenhoek 35, 325–343. 10.1007/BF02219153 5309907

[B11] Hashidoko Y. Itoh E. Yokota K. Yoshida T. Tahara S. (2002). Characterization of five phyllosphere bacteria isolated from *Rosa rugosa* leaves, and their phenotypic and metabolic properties. Biosci. Biotechnol. Biochem. 66, 2474–2478. 10.1271/bbb.66.2474 12506991

[B12] He Z. Wiegel J. (1995). Purification and characterization of an oxygen-sensitive reversible 4-hydroxybenzoate decarboxylase from *Clostridium hydroxybenzoicum*. Eur. J. Biochem. 229, 77–82. 10.1111/j.1432-1033.1995.0077l.x 7744052

[B13] He Z. Wiegel J. (1996). Purification and characterization of an oxygen-sensitive, reversible 3,4-dihydroxybenzoate decarboxylase from *Clostridium hydroxybenzoicum*. J. Bacteriol. 178, 3539–3543. 10.1128/jb.178.12.3539-3543.1996 8655551 PMC178123

[B14] Ibraheem O. Ndimba B. K. (2013). Molecular adaptation mechanisms employed by ethanologenic bacteria in response to lignocellulose-derived inhibitory compounds. Int. J. Biol. Sci. 9, 598–612. 10.7150/ijbs.6091 23847442 PMC3708040

[B15] Jiménez N. Curiel J. A. Reverón I. de Las Rivas B. Muñoz R. (2013). Uncovering the *Lactobacillus plantarum* WCFS1 gallate decarboxylase involved in tannin degradation. Appl. Environ. Microbiol. 79, 4253–4263. 10.1128/AEM.00840-13 23645198 PMC3697502

[B16] Junker A. Rohn H. Czauderna T. Klukas C. Hartmann A. Schreiber F. (2012). Creating interactive, web-based and data-enriched maps using the systems biology graphical notation. Nat. Protoc. 7, 579–593 10.1038/nprot.2012.002 22383037

[B17] Klabunde J. Kunze G. Gellissen G. Hollenberg C. P. (2003). Integration of heterologous genes in several yeast species using vectors containing a *Hansenula polymorpha*-derived rDNA-targeting element. FEMS Yeast Res. 4, 185–193. 10.1016/S1567-1356(03)00148-X 14613883

[B18] Krumholz L. R. Crawford R. L. Hemling M. E. Bryant M. P. (1987). Metabolism of gallate and phloroglucinol in *Eubacterium oxidoreducens* via 3-hydroxy-5-oxohexanoate. J. Bacteriol. 169, 1886–1890. 10.1128/jb.169.5.1886-1890.1987 3571153 PMC212039

[B19] Kunze G. Gaillardin C. Czernicka M. Durrens P. Martin T. Böer E.. (2014). The complete genome of *Blastobotrys* (*Arxula*) *adeninivorans* LS3: a yeast of biotechnological interest. Biotechnol. Biofuels 7:66. 10.1186/1754-6834-7-66 24834124 PMC4022394

[B20] Kunze G. Kunze I. (1994). Characterization of *Arxula adeninivorans* strains from different habitats. Antonie Van Leeuwenhoek 65, 607–614. 10.1007/BF00878276 8060121

[B21] Kunze I. Nilsson C. Adler K. Manteuffel R. Horstmann C. Bröker M.. (1998). Correct targeting of a vacuolar tobacco chitinase in *Saccharomyces cerevisiae*- post-translational modifications are dependent on the host strain. Biochim. Biophys. Acta 1395, 329–344. 10.1016/S0167-4781(97)00163-2 9512669

[B22] Li W. Wang C. (2015). Biodegradation of gallic acid to prepare pyrogallol by *Enterobacter aerogenes* through substrate induction. Bioresources 10, 3027–3044. 10.15376/biores.10.2.3027-3044

[B23] Livak K. J. Schmittgen T. D. (2001). Analysis of relative gene expression data using real-time quantitative PCR and the 2−ΔΔ*C*_T_. method. Methods 25, 402–408. 10.1006/meth.2001.1262 11846609

[B24] Malak A. Baronian K. Kunze G. (2016). *Blastobotrys* (*Arxula*) *adeninivorans*: a promising alternative yeast for biotechnology and basic research. Yeast 33, 535–547. 10.1002/yea.3180 27372304

[B25] Middelhoven W. J. Hoogkamer-Te Niet M. V. Kreger-Van Rij N. J. W. (1984). *Trichosporon adeninovorans* sp. nov., a yeast species utilizing adenine, xanthine, uric acid, putrescine and primary n-alkylamines as the sole source of carbon, nitrogen and energy. Antonie van Leeuwenhoek 50, 369–378. 10.1007/BF00394651 6543110

[B26] Mukherjee G. Banerjee R. (2004). Biosynthesis of tannase and gallic acid from tannin rich substrates by *Rhizopus oryzae* and *Aspergillus foetidus*. J. Basic Microbiol. 44, 42–48. 10.1002/jobm.200310317 14768027

[B27] Nakajima H. Otani C. Niimura T. (1992). Decarboxylation of gallate by cell-free extracts of *Streptococcus faecalis* and *Klebsiella pneumoniae* isolated from rat feces. J. Food Hyg. Soc. Jpn. 33, 371–376. 10.3358/shokueishi.33.371

[B28] Noda F. Hayaski K. Mizunuma T. (1982). Influence of pH on inhibitory activity of acetic acid on osmophilic yeasts used in brine fermentation of soy sauce. Appl. Environ. Microbiol. 43, 245–246. 16345925 10.1128/aem.43.1.245-246.1982 PMC241808

[B29] O'Donovan L. Brooker J. D. (2001). Effect of hydrolysable and condensed tannins on growth, morphology and metabolism of *Streptococcus gallolyticus* (*S. caprinus*) and *Streptococcus bovis*. Microbiology 147, 1025–1033. 10.1099/00221287-147-4-1025 11283298

[B30] Omura H. Wieser M. Nagasawa T. (1998). Pyrrole-2-carboxylate decarboxylase from *Bacillus megaterium* PYR2910, an organic-acid-requiring enzyme. Eur. J. Biochem. 253, 480–484. 10.1046/j.1432-1327.1998.2530480.x 9654100

[B31] Restaino L. Lenovich L. M. Bills S. (1982). Effect of acids and sorbate combinations on the growth of four osmophilic yeasts. J. Food Protection 45, l38–l42. 10.4315/0362-028X-45.12.1138 30913720

[B32] Reverón I. de las Rivas B. Matesanz R. Muñoz R. López de Felipe F. (2015). Molecular adaptation of *Lactobacillus plantarum* WCFS1 to gallic acid revealed by genome-scale transcriptomic signature and physiological analysis. Microb. Cell Fact. 14:160. 10.1186/s12934-015-0345-y 26453568 PMC4600210

[B33] Rodríguez H. Landete J. M. de las Rivas B. Muñoz R. (2008). Metabolism of food phenolic acids by *Lactobacillus plantarum* CECT 748^T^. Food Chem. 107, 1393–1398. 10.1016/j.foodchem.2007.09.067

[B34] Rohn H. Junker A. Czauderna T. Hartmann A. Klapperstück M. Treutler H.. (2012). VANTED v2: a framework for systems biology applications. BMC Systems Biol. 6:139. 10.1186/1752-0509-6-139 23140568 PMC3610154

[B35] Rösel H. Kunze G. (1995). Cloning and characterization of a *TEF* gene for elongation factor 1 alpha from the yeast *Arxula adeninivorans*. Curr. Genet. 28, 360–366. 10.1007/BF00326434 8590482

[B36] Rose M. D. Winston F. Hieter P. (1990). Methods in Yeast Genetics. A Laboratory Manual. Cold Spring Harbor, NY: Cold Spring Harbor Laboratory.

[B37] Sietmann R. Uebe R. Böer E. Bode R. Kunze G. Schauer F. (2010). Novel metabolic routes during the oxidation of hydroxylated aromatic acids by the yeast *Arxula adeninivorans*. J. Appl. Microbiol. 108, 789–799. 10.1111/j.1365-2672.2009.04474.x 19702859

[B38] Sikkema J. de Bont J. A. Poolman B. (1995). Mechanisms of membrane toxicity of hydrocarbons. Microbiol. Rev. 59, 201–222. 7603409 10.1128/mr.59.2.201-222.1995 PMC239360

[B39] Smyth G. K. (2004). Linear models and empirical Bayes methods for assessing differential expression in microarray experiments. Stat. Appl. Genet. Mol. Biol. 3, 3. 10.2202/1544-6115.1027 16646809

[B40] Smyth G. K. (2005). Limma: linear models for microarry data, in Bioinformatics and Computational Biology Solutions Using R and Bioconductor, eds Gentleman R. Carey V. Dudoit S. I. Huber W. (New York, NY: Springer), 397–420.

[B41] Steinborn G. Gellissen G. Kunze G. (2007). A novel vector element providing multicopy vector integration in *Arxula adeninivorans*. FEMS Yeast Res. 7, 1197–1205. 10.1111/j.1567-1364.2007.00280.x 17655689

[B42] Tag K. Lehmann M. Chan C. Renneberg R. Riedel K. Kunze G. (1998). *Arxula adeninivorans* LS3 as suitable biosensor for measurements of biodegradable substances in salt water. J. Chem. Technol. Biotechnol. 73, 385–388.

[B43] Tanaka A. Ohnishi N. Fukui S. (1967). Studies on the formation of vitamins and their function in hydrocarbon fermentation. production of vitamins and their function in hydrocarbon medium. J. Ferment. Technol. 45, 617–623.

[B44] Teste M. A. Duquenne M. François J. M. Parrou J. L. (2009). Validation of reference genes for quantitative expression analysis by real-time RT-PCR in *Saccharomyces cerevisiae*. BMC Mol. Biol. 10, 99–113. 10.1186/1471-2199-10-99 19874630 PMC2776018

[B45] Wartmann T. Böer E. Pico A. H. Sieber H. Bartelsen O. Gellissen G.. (2002). High-level production and secretion of recombinant proteins by the dimorphic yeast *Arxula adeninivorans*. FEMS Yeast Res. 2, 363–369. 10.1111/j.1567-1364.2002.tb00105.x 12702286

[B46] Wartmann T. Krüger A. Adler K. Duc B. Kunze I. Kunze K. (1995). Temperature-dependent dimorphism of the yeast *Arxula adeninivorans* LS3. Antonie Van Leeuwenhoek 68, 215–223. 10.1007/BF00871818 8572679

[B47] Williams P. A. Sayers J. R. (1994). The evolution of pathways for aromatic hydrocarbon oxidation in *Pseudomonas*. Biodegradation 5, 195–217. 10.1007/BF00696460 7765833

[B48] Worch S. Lemke I. (2017). Gene expression analysis in *Arxula adeninivorans*: a nested quantitative real time PCR approach, in Yeast Diversity in Human Welfare, eds Satyanarayana T. Kunze G. (Singapore: Springer Science + Business Media), 251–256.

[B49] Wright J. D. (1993). Fungal degradation of benzoic acid and related compounds. World J. Microbiol. Biotechnol. 9, 9–16. 10.1007/BF00656508 24419831

[B50] Yang X. Wartmann T. Stoltenburg R. Kunze G. (2000). Halotolerance of the yeast *Arxula adeninivorans* LS3. Antonie Van Leeuwenhoek 77, 303–311. 10.1023/A:1002636606282 10959559

[B51] Yoshida H. Tani Y. Yamada H. (1982). Isolation and identification of a pyrogallol producing bacterium from soil. Agric. Biol. Chem. 46, 2539–2546.

[B52] Yoshida H. Yamada H. (1985). Microbial production of pyrogallol through decarboxylation of gallic acid. Agric. Biol. Chem. 49, 659–663.

[B53] Yoshida T. Inami Y. Matsui T. Nagasawa T. (2010). Regioselective carboxylation of catechol by 3,4-dihydroxybenzoate decarboxylase of *Enterobacter cloacae* P. Biotechnol. Lett. 32, 701–705. 10.1007/s10529-010-0210-3 20131080

[B54] Zeida M. Wieser M. Yoshida T. Sugio T. Nagasawa T. (1998). Purification and characterization of gallic acid decarboxylase from *Pantoea agglomerans* T71. Appl. Environ. Microbiol. 64, 4743–4747. 10.1128/aem.64.12.4743-4747.1998 PMC90917 9835557

[B55] Zhang Z. Pang Q. Li M. Zheng H. Chen H. Chen K. (2014). Optimization of the condition for adsorption of gallic acid by *Aspergillus oryzae* mycelia using Box-Behnken design. Environ. Sci. Pollut. Res. 22, 1085–1094. 10.1007/s11356-014-3409-3 25109471

