# Supplementary material for: Agdc1p – a Gallic Acid Decarboxylase Involved in the Degradation of Tannic Acid in the Yeast Blastobotrys (Arxula) adeninivorans
Source: Front Microbiol. 2017 Sep 15;8:1777. doi: 10.3389/fmicb.2017.01777 (PMC5605622; doi:10.3389/fmicb.2017.01777)
Supplement: Supplementary file 1 [file DataSheet1.PDF]

Supplementary Material

Agdc1p – a gallate decarboxylase involved in the degradation of tannic acid in the yeast *Blastobotrys (Arxula) adeninivorans*

Anna Malak[a], Sebastian Worch[a], Erik Böer[a], Anja Hartmann[a], Martin Mascher[a], Marek Marzec[a,b], Uwe Scholz[a], Jan Riechen[a], Kim Baronian[c], Frieder Schauer[d], Rüdiger Bode[d] and Gotthard Kunze[a*]

[a] *Leibniz Institute of Plant Genetics and Crop Plant Research (IPK), Correnstr. 3, D-06466 Gatersleben, Germany*

[b] *Department of Genetics, Faculty of Biology and Environmental Protection, University of Silesia, Katowice 40-032, Poland*

[c] *School of Biological Sciences, University of Canterbury, Private Bag 4800, Christchurch, New Zealand*

[d] *Institute of Microbiology, University of Greifswald, Jahnstr. 15, D-17487 Greifswald, Germany*

* Corresponding author: G. Kunze, Leibniz Institute of Plant Genetics and Crop Plant Research (IPK), Corrensstr. 3, D-06466 Gatersleben, Saxony-Anhalt/Germany. Tel. (+49) 39482-5247; Fax (+49) 39482-5366; e-mail: kunzeg@ipk-gatersleben.de

1. Supplementary Tables

Substrate	K_m [mM]	k_{cat} [s^{-1}]	k_{cat}/K_m [mM^{-1} s^{-1}]
gallic acid	0.7 ± 0.2	42.0 ± 8.2	57.8 ± 7.6
protocatechuic acid	3.2 ± 0.2	44.4 ± 3.2	14.0 ± 2.0

Supplementary Table S1. Kinetic constants of purified Agdc1-6hp synthetized by G1212/YRC102-AYNI1-AGDC1-6H for gallic acid and protocatechuic acid as substrates. Measurement was assayed spectrophotometrically.

1. Supplementary Figures

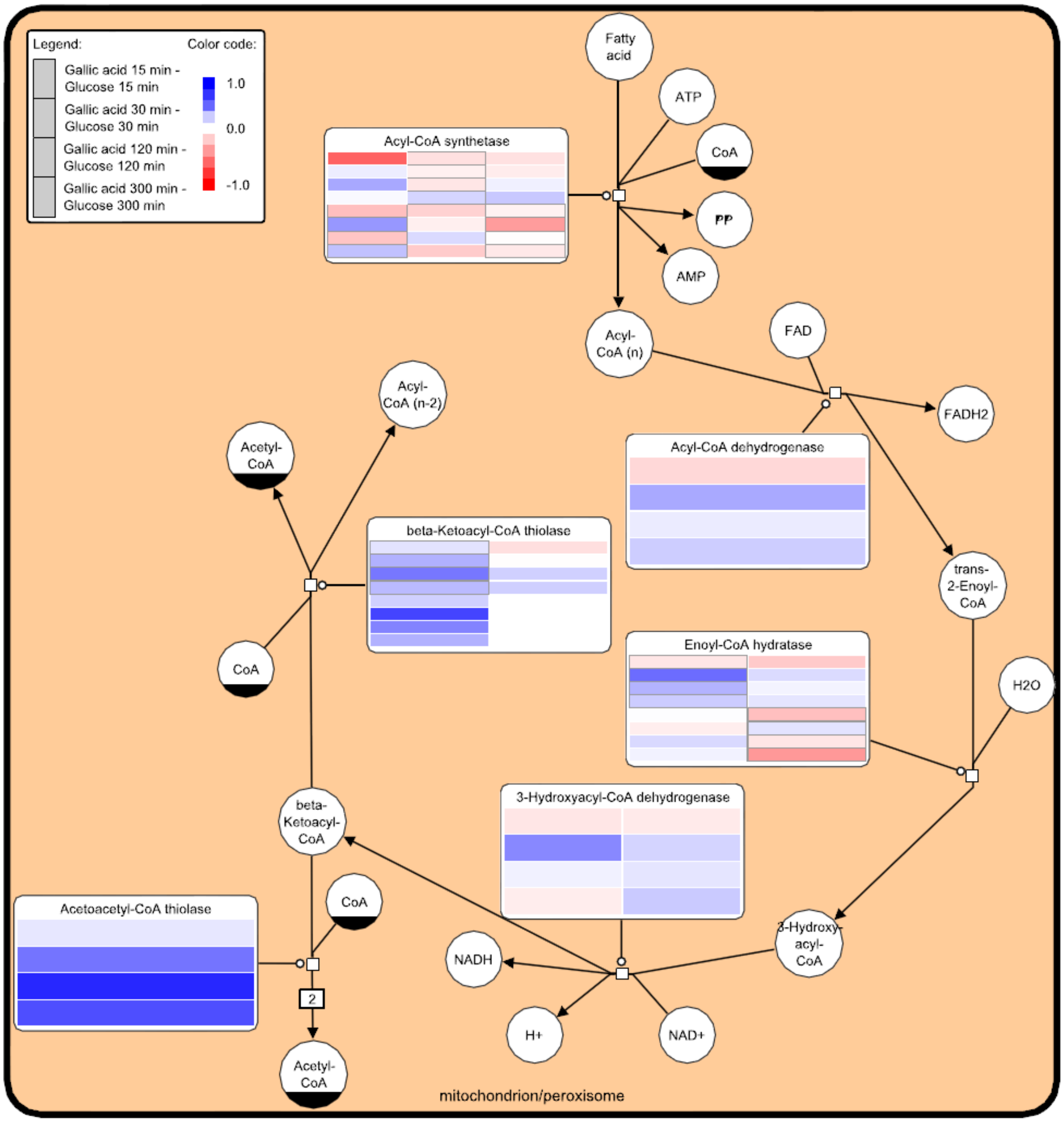

Supplementary Figure S1. Key compounds of the β-oxidation - microarray studies. The SBGN style metabolic network depicts reactions catalyzed by the corresponding enzymes (rectangular square). Enzymes are enriched with color-coded fold change values of time resolved expression data of the respective genes. The colors represent upregulation (blue) and downregulation (red) of genes in cells shifted to a medium containing 0.5% gallic acid and 1% glucose as the carbon sources compared to cells grown with 1% glucose. Metabolites or enzymes occurring multiple times in the metabolic network are decorated with a clone marker (e. g. NAD^+) [produced using VANTED – (Rohn et al., 2012; Junker et al., 2012)].

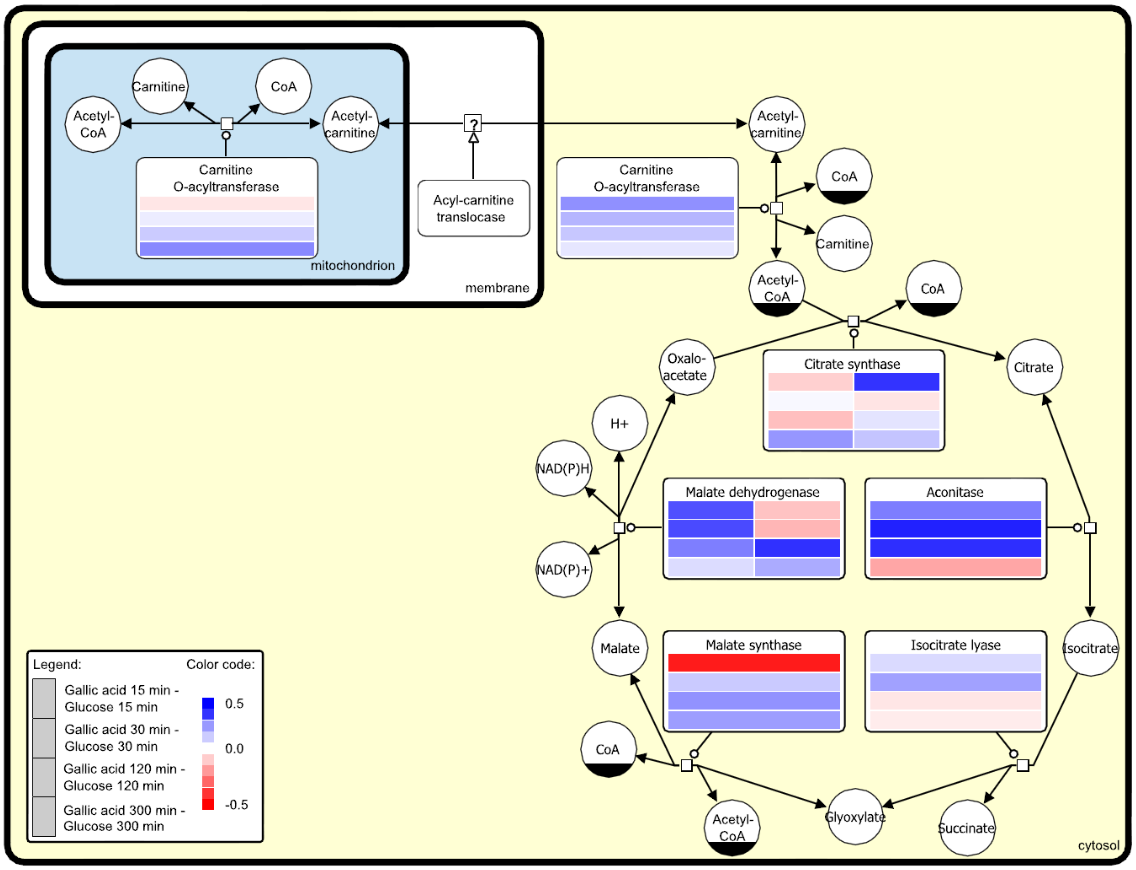

Supplementary Figure S2. Key compounds of the glyoxylate cycle - microarray studies. The SBGN style metabolic network depicts reactions catalyzed by the corresponding enzymes (rectangular square). Enzymes are enriched with color-coded fold change values of time resolved expression data of the respective genes. The colors represent upregulation (blue) and downregulation (red) of genes in cells shifted to a medium containing 0.5% gallic acid and 1% glucose as the carbon sources compared to cells grown with 1% glucose. Metabolites or enzymes occurring multiple times in the metabolic network are decorated with a clone marker (e. g. NAD^+) [produced using VANTED – (Rohn et al., 2012; Junker et al., 2012)].

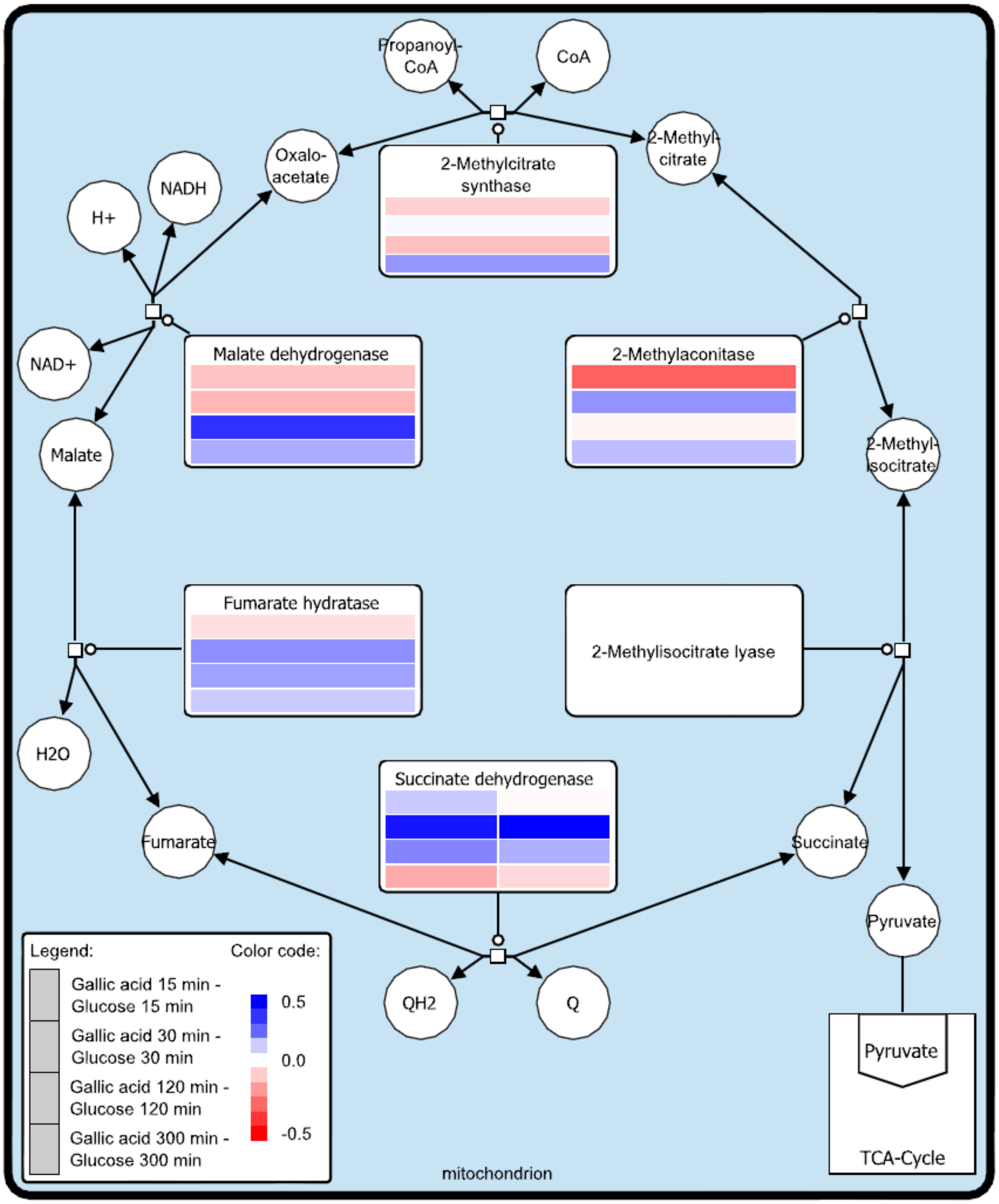

Supplementary Figure S3. Key compounds of the methyl citrate cycle - microarray studies. The SBGN style metabolic network depicts reactions catalyzed by the corresponding enzymes (rectangular square). Enzymes are enriched with color-coded fold change values of time resolved expression data of the respective genes. The colors represent upregulation (blue) and downregulation (red) of genes in cells shifted to a medium containing 0.5% gallic acid and 1% glucose as the carbon sources compared to cells grown with 1% glucose. Metabolites or enzymes occurring multiple times in the metabolic network are decorated with a clone marker (e. g. NAD^+) [produced using VANTED – (Rohn et al., 2012; Junker et al., 2012)].

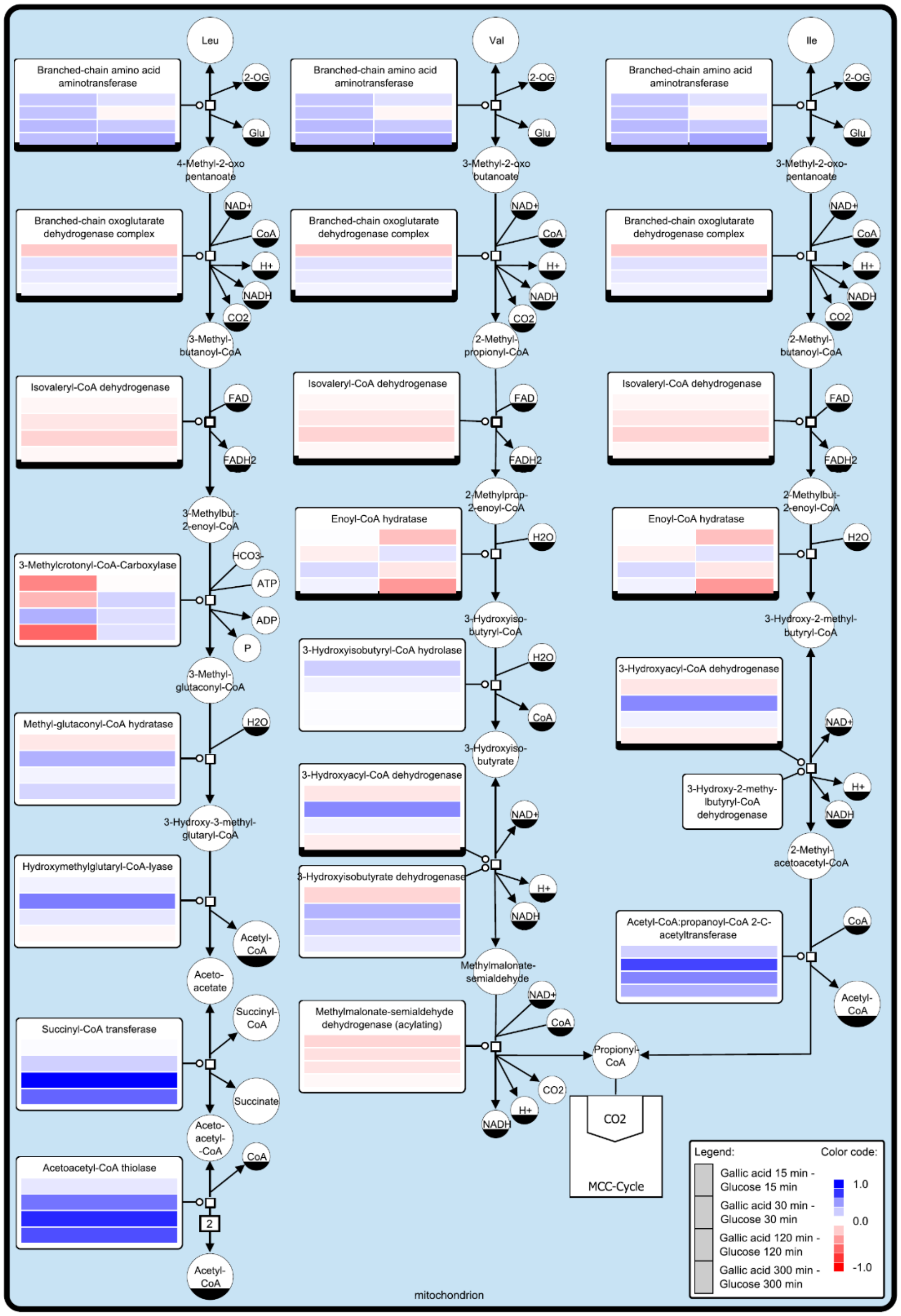

Supplementary Figure S4. Key compounds of the catabolism of the branched-chain amino acids valine, leucine and isoleucine - microarray studies. The SBGN style metabolic network depicts reactions catalyzed by the corresponding enzymes (rectangular square). Enzymes are enriched with color-coded fold change values of time resolved expression data of the respective genes. The colors represent upregulation (blue) and downregulation (red) of genes in cells shifted to a medium containing 0.5% gallic acid and 1% glucose as the carbon sources compared to cells grown with 1% glucose. Metabolites or enzymes occurring multiple times in the metabolic network are decorated with a clone marker (e. g. NAD^+) [produced using VANTED – (Rohn et al., 2012; Junker et al., 2012)].

(A)

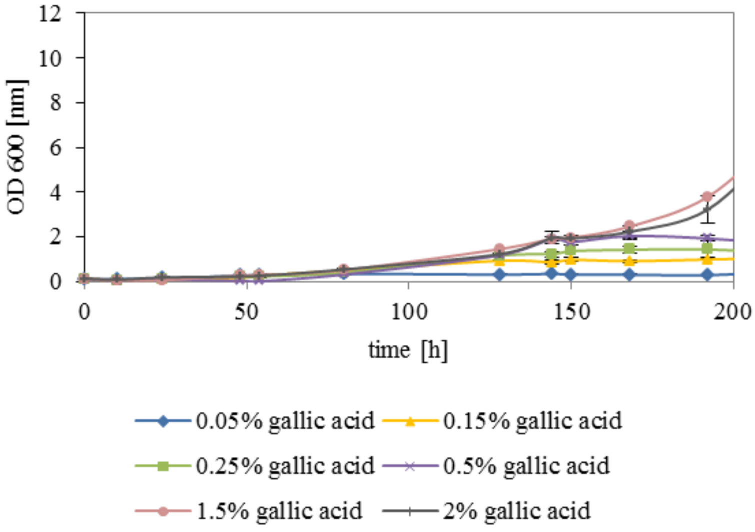

(B)

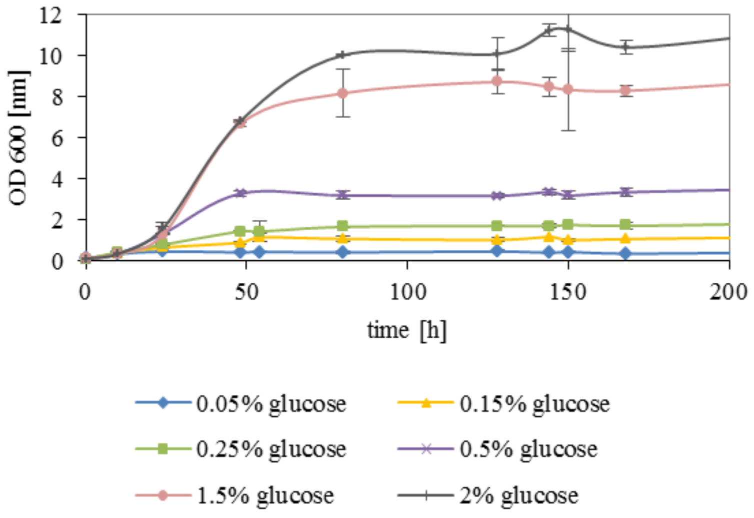

Supplementary Figure S5. (**A**) Gallic acid tolerance of *A. adeninivorans* G1212/YRC102. The strain was cultivated on YMM-$NaNO_3$ with different concentrations of gallic acid. (**B**) As control *A. adeninivorans* G1212/YRC102 was cultivated on YMM-$NaNO_3$ with corresponding concentrations of glucose.
